# Tumor Cell Derived Lnc‐FSD2‐31:1 Contributes to Cancer‐Associated Fibroblasts Activation in Pancreatic Ductal Adenocarcinoma Progression through Extracellular Vesicles Cargo MiR‐4736

**DOI:** 10.1002/advs.202203324

**Published:** 2023-02-02

**Authors:** Xinglong Geng, Le Li, Yan Luo, Wenbo Yang, Jisheng Hu, Zhongjie Zhao, Chundong Cheng, Tao Zhang, Yangyang Zhang, Liwei Liu, Yu Xie, Guanqun Li, Danxi Liu, Rui Bai, Xuewei Bai, Gang Wang, Hua Chen, Yongwei Wang, Hongze Chen, Bei Sun

**Affiliations:** ^1^ Department of Pancreatic and Biliary Surgery The First Affiliated Hospital of Harbin Medical University Key Laboratory of Hepatosplenic Surgery Ministry of Education The First Affiliated Hospital of Harbin Medical University Harbin Heilongjiang 150000 China

**Keywords:** autophagy, cancer‐associated fibroblasts, extracellular vesicles, noncoding RNA, pancreatic ductal adenocarcinoma

## Abstract

Pancreatic ductal adenocarcinoma (PDAC) presents with high mortality and short overall survival. Cancer‐associated fibroblasts (CAFs) act as refuge for cancer cells in PDAC. Mechanisms of intracelluar communication between CAFs and cancer cells need to be explored. Long noncoding RNAs (lncRNAs) are involved in the modulation of oncogenesis and tumor progression of PDAC; however, specific lncRNAs and their mechanism of action have not been clarified clearly in tumoral microenvironment. This work aims to identify novel lncRNAs involved in cellular interaction between cancer cells and CAFs in PDAC. To this end, differentially expressed lncRNAs between long‐term and short‐term survival PDAC patients are screened. Lnc‐FSD2‐31:1 is found to be significantly increased in long‐term survival patients. This work then discovers that tumor‐derived lnc‐FSD2‐31:1 restrains CAFs activation via miR‐4736 transported by extracellular vesicles (EVs) in vitro and in vivo. Mechanistically, EVs‐derived miR‐4736 suppresses autophagy and contributes to CAFs activation by targeting ATG7. Furthermore, blocking miR‐4736 suppresses tumor growth in genetically engineered KPC (LSL‐KrasG12D/+, LSL‐Trp53R172H/+, and Pdx‐1‐Cre) mouse model of PDAC. This study demonstrates that intratumoral lnc‐FSD2‐31:1 modulates autophagy in CAFs resulting in their activation through EVs‐derived miR‐4736. Targeting miR‐4736 may be a potential biomarker and therapeutic target for PDAC.

## Graphical abstract

This work reports a downregulated lncRNA, lnc‐FSD2‐31:1, which is featured as tumor suppressor and sentry duty of screening pancreatic ductal adenocarcinoma (PDAC) patient's outcome. Cancer cell‐derived lnc‐FSD2‐31:1 releases miR‐4736 through extracellular vesicles (EVs) cargo that regulates autophagy and fibrotic phenotypes in cancer‐associated fibroblasts (CAFs). This study discovers a framework of understanding EVs‐dependent communications between cancer cells and CAFs in PDAC.

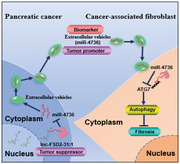

## 1 Introduction

Pancreatic cancer (PCa) is known as one of the most lethal malignant tumors and the 5‐year post survival rate is less than 10%.^[^ ^1^ ^]^ PCa presents with complex tissue organization with variety of different cell types in tumor microenvironment (TME), including cancer cells, fibroblast, and cell–cell interactions.^[^ ^2^ ^]^ Pancreatic ductal adenocarcinoma (PDAC) is the most prevalent form of PCa and its tumorigenesis is associated with inactivated tumor suppressing genes, such as *P53*, *CDKN2A/p16*, combined with *KRAS* mutation.^[^ ^3^ ^]^ Recent studies indicate that long noncoding RNAs (lncRNAs) are involved in multiple aspects of PDAC tumorigenesis and development;^[^ ^4^ ^]^ for instance, they provide scaffolding structure to regulate tumor proliferation,^[^ ^5^ ^]^ interact with RNA binding proteins (RBP) to remodel malignant phenotypes,^[^ ^6^ ^]^ and sponge and sequester miRNAs to affect tumoral properties.^[^ ^7^ ^]^ Therefore, lncRNA may play a key role in the progression of PDAC.

Extracellular vesicles (EVs) act as intercellular signaling compartments, which can transport proteins and nucleotides^[^ ^8^ ^]^ to recipient cells to mediate phenotypic alterations.^[^ ^9^ ^]^ EV‐derived lncRNAs *HULC*,^[^ ^10^ ^]^ *MALAT1*,^[^ ^11^ ^]^ and *KRT19* ^[^ ^12^ ^]^ have been shown to affect PDAC progression. Furthermore, EV‐derived miR‐132 could affect PDAC proliferation through Pten/Akt/Foxo3 signaling.^[^ ^13^ ^]^ In addition, EV‐derived miR‐181a‐5p could affect hepatic stellate cells activation to promote liver metastasis in colorectal cancer.^[^ ^14^ ^]^ It was shown that EV‐derived miR‐630 could affect cancer‐associated fibroblasts (CAFs) activation to facilitate invasion and metastasis of ovarian cancer.^[^ ^15^ ^]^ Stroma forms up to 90% of PDAC volume, and CAFs represent its dominant component.^[^ ^16^ ^]^ CAFs can enhance stromal stiffness by producing collagen; furthermore, they secrete chemokines and cytokines (e.g., IL‐1 and IL‐6), and alter the immune cell milieu by recruiting immune suppressive cells.^[^ ^17^ ^]^ However, the mechanisms by which tumor cell‐derived EVs regulate CAFs activation remain unclear in PDAC. Here, we aimed to investigate whether novel lncRNAs in PDAC are involved in CAFs activation with EVs being mediators in the cellular interaction.

## 2 Results

### 2.1 Overexpression of Lnc‐FSD2‐31:1 Predicts Long‐Term Survival in PDAC

**Figure 1:**
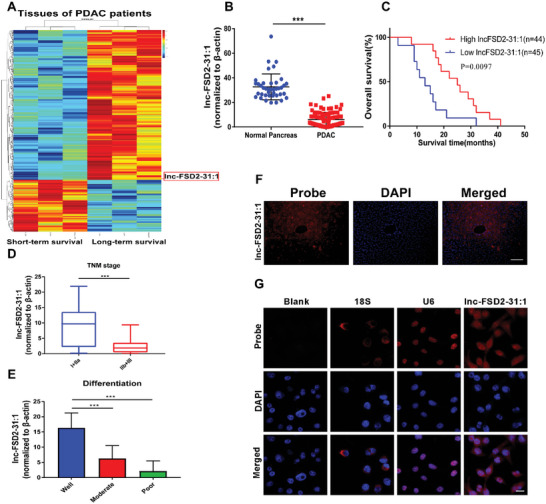
Overexpression of Lnc‐FSD2‐31:1 predicts long‐term survival in pancreatic ductal adenocarcinoma (PDAC). A) Heatmap of lncRNAs between long‐term survival and short‐term survival PDAC patient tissues. (*n* = 3, long‐term survival >5 years, short‐term survival <6 months). Total of 489 lncRNAs are identified, different RNAs are screened according to the criteria of log2FC > 2 and *p*‐value < 0.05. Duplicate RNAs, coding genes (https://lncipedia.org/), and genes less than 200 bp are filtered out. The studied lncRNAs are excluded. After the initial screening, the remaining lncRNAs are sequenced according absolute foldchange. At last, the “top 10” discrepant lncRNA expressions are tested in 89 PDAC and 40 cancer adjacent tissues using quantitative real‐time polymerase chain reaction (qRT‐PCR). The lnc‐FSD2‐31:1 is particularly prominent in tumor progression. B) qRT–PCR assays detect the expression of lnc‐FSD2‐31:1 in 89 PDAC and 40 cancer adjacent tissues. C) Kaplan–Meier plot shows overall survival on patients with PDAC. The high and low levels of lnc‐FSD2‐31:1 expression are separated according to the median value. Kaplan–Meier survival analysis is used to evaluate overall survival and statistical significance is assessed using Student's *t* test. D) Correlation analysis between levels of lnc‐FSD2‐31:1 expression and TNM stage of PDAC. E) Correlation analysis between levels of lnc‐FSD2‐31:1 expression and differentiation of PDAC. F) RNA fluorescence in‐situ hybridization (FISH) images show the location of lnc‐FSD2‐31:1 in the PDAC tissue. (Original magnification, 10×, scale bar, 100 µm). G) RNA FISH images show the location of lnc‐FSD2‐31:1 in the nuclei and cytoplasm of PANC‐1 cells. 18S, probe for 18S rRNA; U6, probe for U6 snRNA. (Original magnification, 40×, scale bar, 20 µm). Statistical significance is assessed using Student's *t* test. Data are shown as the mean ± SD of three replicates. ^***^ *p* < 0.001.

Transcriptome sequencing was used to investigate the differentially expressed lncRNAs between long‐term (>5 years) and short‐term survival (<6 months) PDAC patients, and a total of 489 lncRNAs were identified (**Figure** **1**A, Figure S1A, Supporting Information). The “top 10” discrepant lncRNA expressions were tested in 89 PDAC and 40 cancer adjacent tissues using quantitative real‐time polymerase chain reaction (qRT‐PCR) (Figure 1B, Figure S1B, Supporting Information). We found that a long‐term survival related lncRNA, named lnc‐FSD2‐31:1, was particularly prominent in tumor progression (Figure 1B). The noncoding property of lnc‐FSD2‐31:1 was confirmed by coding‐potential analysis (Figure S1C,D, Supporting Information).^[^ ^18^ ^]^ Patients with higher level of lncFSD2‐31:1 exhibited better overall survival (OS) (Figure 1C). Further analysis indicated that lnc‐FSD2‐31:1 expression was negatively correlated with TNM stage and differentiation of PDAC (Figure 1D,E). To clarify the presence and the localization of lnc‐FSD2‐31:1, RNA fluorescence in situ hybridization (FISH) was performed. Our results indicated that lnc‐FSD2‐31:1 was localized in the nucleus and cytoplasm in tumor cells (Figure 1F,G). Cumulatively, lnc‐FSD2‐31:1 is consistent with our expectation of project design and these results suggest that tumor cell‐derived lnc‐FSD2‐31:1 may be negatively correlated with PDAC development.

### 2.2 Tumor‐Derived Lnc‐FSD2‐31:1 Restrains CAFs Activation Through EVs

**Figure 2:**
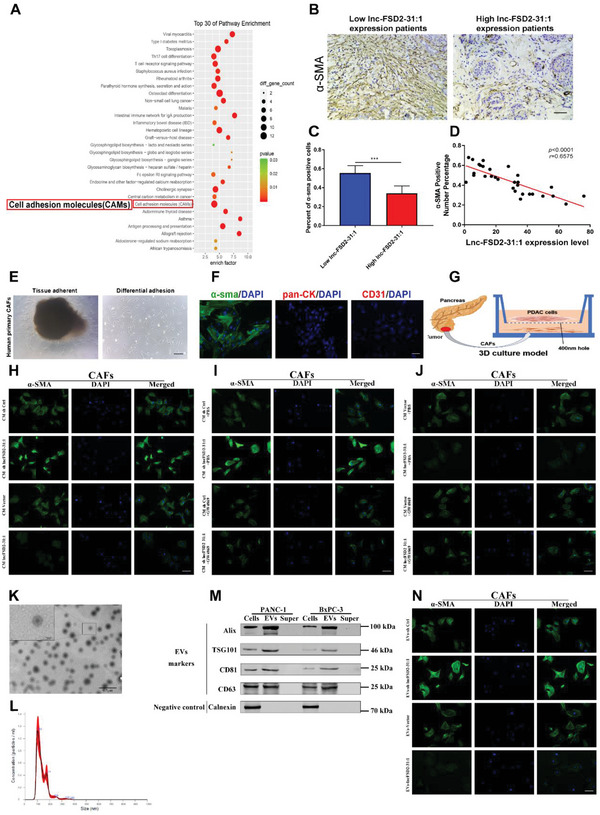
Tumor‐derived lnc‐FSD2‐31:1 restrains cancer‐associated fibroblasts (CAFs) activation through extracellular vesicles. A) KEGG pathway analysis is performed on the transcriptome sequencing of differentially expressed mRNAs between long‐term and short‐term survival pancreatic ductal adenocarcinoma (PDAC) patients. Cell adhesion molecules (CAMs) pathways are closely related to the dense stroma characteristics of PDAC, which is one of the most genetically enriched pathways in the KEGG. B–D) Correlation analysis between lnc‐FSD2‐31:1 expression level and *α*‐SMA positive percentage of immunohistochemical (IHC) sections of pancreatic tumors. Negative correlation between lnc‐FSD2‐31:1 and *α*‐SMA expression in pancreatic tumors (Original magnification, 20×, scale bar, up 50 µm). E) Primary CAFs are isolated from PDAC patient pancreatic cancer using the enzyme digestion method (Original magnification, 20×, Scale bar, 50 µm). F) Immunofluorescence (IF) images show that isolated CAFs are positive for *α*‐SMA and negative for pancytokeratin and CD‐31 (original magnification, 20×, scale bar, 50 µm). G) The monoculture system is established to coculture PANC‐1 cells and CAFs. There is a membrane separating the two types of cells, and they communicate through small holes in the membrane of less than 400 nm. H) IF detection of *α*‐SMA expression in CAFs cultured with conditioned medium (CM) from PANC‐1 cells with different lnc‐FSD2‐31:1 expression levels (original magnification, 20×, scale bar, 50 µm). I‐J) IF detection of *α*‐SMA expression in CAFs cultured with PBS/GW4869 and CM from PANC‐1 cells with different lnc‐FSD2‐31:1 expression level (original magnification, 20×, scale bar, 50 µm). K) Transmission electron microscope (TEM) of purified extracellular vesicles (EVs) derived from PDAC cells (scale bar, 0.5 µm/100 nm). L) The size and concentration of EVs are detected using nanoparticle tracking analysis. M) Western blotting analysis of the EV markers Alix, TS101, CD81, CD63, and negative control calnexin in PDAC cells, EVs, and ultracentrifuged supernatant. N. IF detection of *α*‐SMA expression in CAFs cultured with EVs from pancreatic cancer with different lnc‐FSD2‐31:1 expression level (original magnification, 20×, scale bar, 50 µm).

In order to explore the downstream and the underlying mechanism of lncFSD2‐31:1 in PDAC progression, KEGG pathway analysis was performed on the transcriptome sequencing of differential expressed mRNAs between long‐term and short‐term survival PDAC patients (the number of different genes ≥2, and *p*‐value <0.05). The results showed that the pathways with the highest number of enriched genes were included toxoplasmosis, osteoclast differentiation, cell adhesion molecules (CAMs), Th17 cell differentiation, and other pathways (**Figure** **2**A). PDAC has been distinguished from other tumors by the abundance of stroma, with dense stroma recognized as a crucial mediator of PDAC progression.^[^ ^17a^ ^]^ Meanwhile, we found that CAMs pathways were closely related to the dense stroma characteristics of PDAC. Among the enriched CAMs, *ICAM1*,^[^ ^19^ ^]^ *ITGA8*,^[^ ^20^ ^]^ *CD99*,^[^ ^21^ ^]^ *JAM2*,^[^ ^22^ ^]^ *GLG1*,^[^ ^23^ ^]^ *CDH1*,^[^ ^24^ ^]^ and *OCLN*,^[^ ^25^ ^]^ have been shown to participate in stroma or fibroblasts activation.^[^ ^26^ ^]^ Therefore, we asked whether lnc‐FSD2‐31:1 was involved in CAFs activation. To test this hypothesis, we explored the correlation between lnc‐FSD2‐31:1 level and CAFs activation. To this end, we tested the expression of *α*‐SMA, which is a classical marker of CAFs activation.^[^ ^17b^ ^]^ Our data showed that lnc‐FSD2‐31:1 expression was negatively correlated with *α*‐SMA positive percentage (Figure 2B–D). To link lnc‐FSD2‐31:1 and CAFs activation, primary CAFs isolated from PDAC patient tissues were validated, and a monoculture system including tumor cells and CAFs was established (Figure 2E–G, Figure S2A, Supporting Information).^[^ ^17c^ ^]^ After two cell types were incubated together, the expression level of *α*‐SMA in CAFs was significantly negatively correlated with the expression level of lnc‐FSD2‐31:1 in tumor cells (Figure 2H, Figure S2B–D, Supporting Information). Interestingly, the expression levels of vimentin and podoplanin, which also are the markers of CAFs in PDAC, did not change under the same coculture conditions (Figure S2E,F, Supporting Information).^[^ ^27^ ^]^ Since tumor cells can only make contacts with CAFs through micropores (less than 400 nm), we speculated that EVs may act as carriers to enable the communication between these two cell types. Our data conformed that EVs inhibitor GW4869 could rescue the effects induced by lnc‐FSD2‐31:1 upregulation (Figure 2I,J). We then isolated and validated EVs from tumor cells by ultracentrifugation and cocultured them with CAFs, which generated consistent results (Figure 2K–N). In addition, EdU assays showed that EVs from upregulated lnc‐FSD2‐31:1 tumor cells inhibited CAFs growth (Figure S2G,H, Supporting Information). These findings indicate that tumor cells‐derived lnc‐FSD2‐31:1 restrains CAFs activation and suppresses tumor progression through EVs.

### 2.3 Cancer Cells‐Derived EVs Restrains CAFs Activation via lnc‐FSD2‐31:1‐miR‐4736‐ATG7 Axis

**Figure 3:**
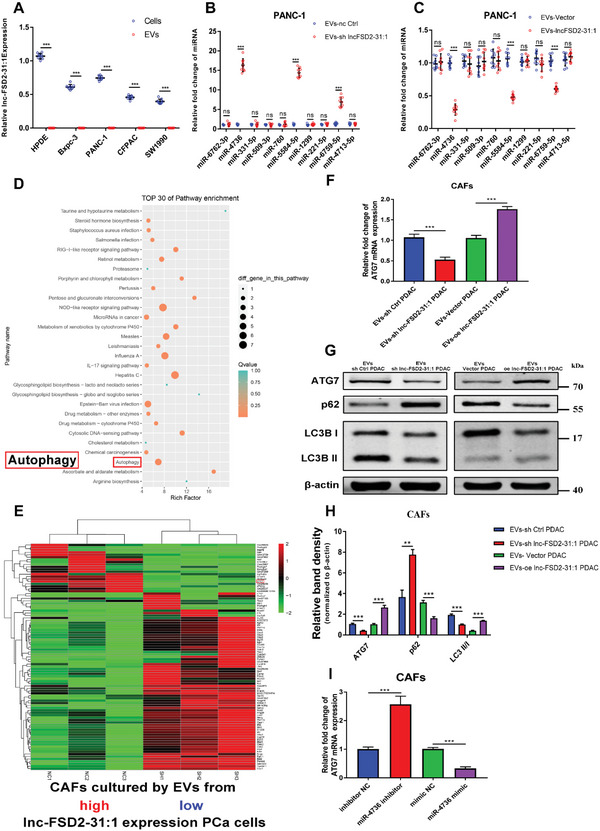
Cancer cells‐derived extracellular vesicles (EVs) restrain cancer‐associated fibroblasts (CAFs) activation via lnc‐FSD2‐31:1‐miR‐4736‐ATG7 axis. A) quantitative real‐time polymerase chain reaction (qRT–PCR) shows that there is rarely lnc‐FSD2‐31:1 in EVs of pancreatic ductal adenocarcinoma (PDAC) compared to cells. B,C) Detecting the expression levels of 10 miRNAs in EVs from pancreatic cancer cells with different lnc‐FSD2‐31:1 expression levels showed that 3 miRNAs (miR‐4736, miR‐5584‐5p, and miR‐6759‐5p) are negatively correlated with lnc‐FSD2‐31:1 expression. D) KEGG pathway analysis is performed on the transcriptome of differentially expressed mRNAs in CAFs, which are cocultured with EVs derived from lnc‐FSD2‐31:1 ‐high/‐low tumor cells. E) Heatmap of mRNA sequencing. Three pairs of CAFs cocultured with EVs from PANC‐1 cells with different lnc‐FSD2‐31:1 expression levels are analyzed. F) Relative fold change in ATG7 mRNA expression in CAFs cultured with EVs from PANC‐1 cells with different lnc‐FSD2‐31:1 expression levels. G,H) Western blot analysis of ATG7, LC3B II, LC3B I, and p62 expression in CAFs cocultured with EVs from PANC‐1 cells with different lnc‐FSD2‐31:1 expression levels. I) qRT–PCR shows that ATG7 mRNA is decreased/increased after transfection of CAFs with miR‐4736 mimic/inhibitor. Statistical significance is assessed using Student's *t* test. Data are shown as the mean ± SD of three replicates; ^***^ *p* < 0.001; ns: not significant.

To clarify the relationship between lnc‐FSD2‐31:1 and EVs, the expression of lnc‐FSD2‐31:1 in EVs was tested revealing the absence of lnc‐FSD2‐31:1 in these structures (**Figure** **3**A). This result suggests that activation of CAFs by cancer cells‐derived EVs was not mediated through endocytosis of lnc‐FSD2‐31:1. EVs‐delivered miRNAs during cancer cell communication have been regarded as crucial and complex processes that reshape TME.^[^ ^28^ ^]^ Consequently, we employed the lncRNASNP2 database to predict the miRNAs that might bind to lnc‐FSD2‐31:1 and 58 miRNAs were identified. Ten of them were validated using qRT‐PCR, and three miRNAs, including miR‐4736, miR‐5584‐5p, and miR‐6759‐5p, could bind to lnc‐FSD2‐31:1 (Figure 3B,C). To identify the targets in CAFs that are influenced by lnc‐FSD2‐31:1 from cancer cells, KEGG pathway analysis was performed on the transcriptome sequencing of discrepant mRNAs in CAFs (the number of different genes ≥2, and *p*‐value <0.05), which were cocultured with EVs derived from lnc‐FSD2‐31:1‐high/‐low tumor cells. Among the pathways with the most enriched genes in KEGG pathway analysis, autophagy pathway^[^ ^29^ ^]^ was closely related to tumor progression (other pathways were included NOD‐like receptor signaling,^[^ ^30^ ^]^ influenza A,^[^ ^31^ ^]^ hepatitis C,^[^ ^32^ ^]^ Epstein‐Barr virus infection,^[^ ^33^ ^]^ and measle,^[^ ^34^ ^]^) (Figure 3D). Our previous studies have proved that autophagy was closely related to PDAC progression.^[^ ^35^ ^]^ Comparing log2FC among the identified 93 mRNAs in transcriptome sequence, mRNA of the ATG7, a key autophagic gene, was found to be the most significant differential expressed mRNA (log2FC = 8.82) (Figure 3E).^[^ ^36^ ^]^ Therefore, we hypothesis that lnc‐FSD2‐31:1 may regulate autophagy in CAFs via ATG7. Meanwhile, all the mRNAs enriched in autophagy pathway were verified via qRT–PCR, and only ATG7 mRNA expression in CAFs was positively correlated with the lnc‐FSD2‐31:1 level in tumor cells (Figure 3F, Figure S3A, Supporting Information). Further, the levels of autophagy were proved to be positively correlated with lnc‐FSD2‐31:1 expression (Figure 3G,H). The TargetScan database was used to predict the candidate miRNAs that might bind to ATG7, and miR‐4736, present in the EVs, was found to bind to lnc‐FSD2‐31:1. We then manipulated the miR‐4736 expression in CAFs and found that higher levels of miR‐4736 resulted in decreased ATG7 expression (Figure 3I, Figure S3B,C, Supporting Information). Taken together, higher or lower lnc‐FSD2‐31:1 in tumor cells activate/decrease CAFs autophagy through modulating EVs‐derived miR‐4736.

### 2.4 MiR‐4736 Targets ATG7 and Restrains Autophagy in CAFs

**Figure 4:**
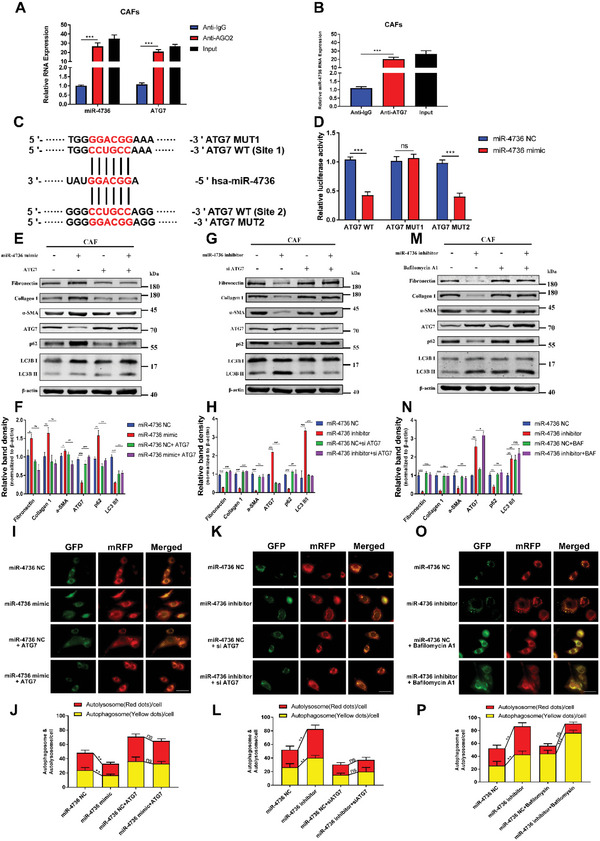
MiR‐4736 targets ATG7 and restrains autophagy in cancer‐associated fibroblasts (CAFs). A,B) RNA immunoprecipitation (RIP) assay in CAFs reveals that miR‐4736 and ATG7 are enriched in anti‐AGO2 pellets compared with anti‐IgG pellets, and miR‐4736 is enriched in anti‐ATG7 pellets compared with anti‐IgG pellets. C) The TargetScan and Rtips databases predict two potential binding sites in ATG7 mRNA that bind to miR‐4736. D) Relative luciferase activity array in CAFs show that the miR‐4736 mimic lower the luciferase activity of pGL3‐ATG7 WT and pGL3‐ATG7 MUT2 but not pGL3‐ATG7 MUT1. E,F) MiR‐4736 mimic and ATG7 overexpression plasmids are cotransfected into CAFs for 48 h, and the cell lysates are subjected to Western blotting. The relative band density is calculated and recorded. MiR‐4736 mimic increases fibrosis levels and decreased autophagy levels in CAFs, and the ATG7 overexpression plasmid rescues these effects. G,H) MiR‐4736 inhibitor and siATG7 are cotransfected into CAFs for 48 h, and the cell lysates are subjected to Western blotting. The relative band density is calculated and recorded. MiR‐4736 inhibitor decreased fibrosis levels and increased autophagy levels of CAFs, and ATG7 siRNA rescued these effects. I–L) The immunofluorescence assays are performed in CAFs that are transfected with flag‐tagged mRFP‐GFP‐LC3 lentiviral vector in four different groups (original magnification, 40×, scar bars, 75 µm). The numbers of GFP and mRFP dots are determined by fluorescent puncta in five high‐power fields. M–P) CAFs are transfected with miR‐4736 inhibitor/NC for 48 h, and interfered with Bafilomycin A1 for 6 h. Western blotting and immunofluorescence assays are used to detect fibrosis levels and autophagic flux in CAFs. Statistical significance is assessed using Student's *t* test. Data are shown as the mean ± SD of three replicates; ^*^ *p* < 0.05; ^**^ *p* < 0.01; ^***^ *p* < 0.001; ns: not significant.

The RNA immunoprecipitation (RIP) assay and the dual luciferase reporting assay were conducted to determine whether miR‐4736 activates CAFs by targeting ATG7. Our data revealed that miR‐4736 and ATG7 were obviously enriched in anti‐AGO2 pellets (**Figure** **4**A). Furthermore, miR‐4736 was enriched in anti‐ATG7 pellets compared with anti‐IgG pellets (Figure 4B). Targetscan and Rtips were employed to predict two potential binding sites between miR‐4736 and ATG7 mRNA 3’UTR (Figure 4C). The dual luciferase reporter assays suggested that only the prediction site 1 of ATG7 could bind to miR‐4736 (Figure 4D). Subsequently, our data suggested that miR‐4736 inhibited autophagy and activated CAFs (Figure 4E–H, Figure S4A,B, Supporting Information). In addition, we performed immunofluorescence (IF) staining and the same results were obtained (Figure S4C,D, Supporting Information). However, when we used vimentin and podoplanin as markers of CAFs, the Western blot assays showed that miR‐4736 could not activate CAFs (Figure S4E,F, Supporting Information). To test whether miR‐4736‐ATG7 axis is able or not to restrain autophagy, a tandem labeled GFP‐mRFP‐LC3 reporter was used in CAFs to measure autophagic flux. Since autophagic flux is a dynamic process, it is imperative to distinguish between the enhanced autophagosome formation and the decreased autophagosome clearance. The GFP‐mRFP‐LC3 staining could be used to localize and assess autophagic flux, which is not limited by pH changes in the cellular environment. The GFP signal is sensitive to the acidic condition of the lysosome lumen, whereas mRFP is much more stable. The colocalization of both GFP and mRFP fluorescent signals indicates an autophagosome or phagophore that was not fused with a lysosome. As shown in Figure 4I,J, both the GFP/RFP and RFP dots were significantly reduced in the miR‐4736 mimic groups and were rescued in ATG7 overexpression groups, while the ratio of GFP/RFP dots was not much different between each group. In contrast, miR‐4736 inhibitor and siATG7 showed the opposite effects (Figure 4I–L). These data demonstrated that miR‐4736‐ATG7 axis decreased the autophagosome formation but not the autophagosome degradation to restrain autophagy in CAFs. In addition, Bafilomycin A1, an inhibitor of lysosomal acidification and fusion with autophagosome, was introduced to block LC‐3 lysosomal degradation and to investigate whether there was autophagosome accumulation in miR‐4736‐ATG7 axis regulation. As shown in the results, Bafilomycin A1 simultaneously accumulated the GFP/RFP dots, decreased the mRFP dots, blocked the lysosomal degradation, and also eliminated the influence of miR‐4736 inhibitor on autophagy (Figure 4M–P, Figure S4G, Supporting Information). Furthermore, the amount of autophagosomes was negatively correlated with miR‐4736 expression in CAFs via TEM (Figure S4H, Supporting Information). Collectively, these results demonstrate that miR‐4736 directly targets ATG7 to restrain autophagosomes formation and activate fibrosis in CAFs.

### 2.5 Lnc‐FSD2‐31:1 Inhibits PDAC Growth In Vitro and In Vivo

**Figure 5:**
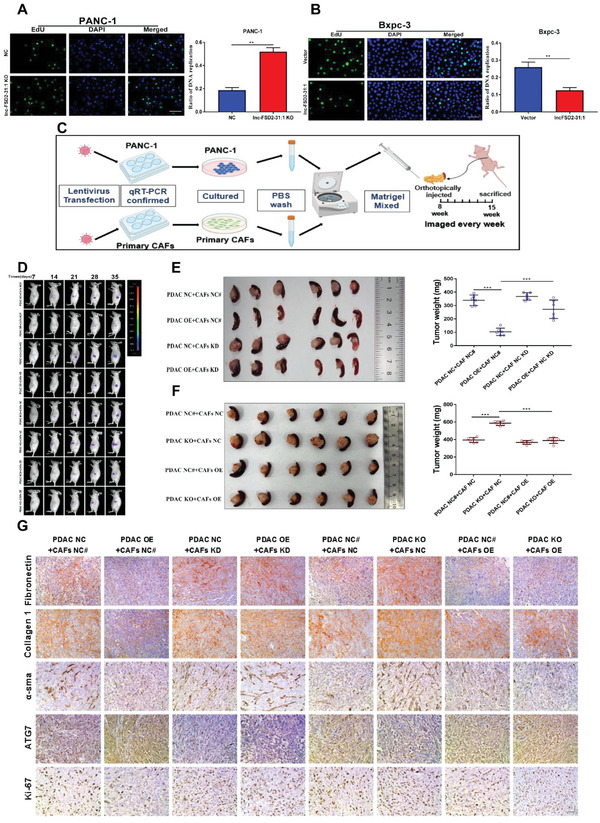
Lnc‐FSD2‐31:1 inhibits pancreatic ductal adenocarcinoma (PDAC) growth in vitro and in vivo. A,B) The proliferative capacity is determined using EdU retention assays in PANC‐1 and BxPC‐3 cells, which are transfected with lentiviruses scramble sgRNA to knockout lnc‐FSD2‐31:1 (LV‐ko lnc‐FSD2‐31:1) and lentiviral vector encoding lnc‐FSD2‐31:1 (LV‐lnc‐FSD2‐31:1) as well as their controls. The ratio of DNA replication in each group is calculated (Original magnification, 20×, bar, 50 µm). C) Schematic diagram displays the process of orthotopic xenograft PDAC model establishment. Through lentivirus transfection, PANC‐1 cells that express lnc‐FSD2‐3:1, and cancer‐associated fibroblasts (CAFs) that express ATG7 differently at a ratio of 1:3 are orthotopically injected into nude mice in different cohorts. D–F) Orthotopic xenograft models are established. In the first cohort, lnc‐FSD2‐31:1 NC or OE PANC‐1 cells with ATG7 NC# or KD CAFs are coinjected at a 3:1 ratio into orthotopic model. In the second cohort, lnc‐FSD2‐31:1 NC# or KO PANC‐1 cells with ATG7 NC or OE CAFs are coinjected at a 3:1 ratio into orthotopic model. Representative bioluminescence imaging (following intraperitoneal injection of 0.1 mg g^−1^ luciferin) of mice at days 7, 14, 21, 28, and 35. At day 35, all mice (*n* = 48, 6 per group, eight groups) are sacrificed, and primary tumors are removed. The weight of tumors in each group is quantified and calculated. Compared to the PDAC NC+CAF NC# group, the tumors of the PDAC OE+CAF NC# group are increased relatively slowly. High lnc‐FSD2‐31:1 expression in PDAC inhibits tumor growth and decreases the weight of tumors. These inhibitory effects disappear when ATG7 is suppressed in CAFs. Compared to the PDAC NC#+CAF NC group, tumor growth in PDAC KO+CAF NC group is increased relatively faster. Low lnc‐FSD2‐31:1 expression in PDAC promotes tumor growth and increases the weight of tumors. These promotion effects disappear when ATG7 is overexpressed in CAFs. G) The expression of fibronectin, collagen 1, *α*‐SMA, ATG7, and Ki67 is detected using IHC in orthotopic tumor specimens (Original magnification, 20×, scale bar, up 50 µm). Statistical significance is assessed by Student's *t* test. Data are shown as the mean ± SD of three replicates; ^**^ *p* < 0.01; ^***^ *p* < 0.001; ns: not significant.

To explore the effects of lncFSD2‐31:1 on tumor growth, cell growth assays were performed, which showed that depletion of lnc‐FSD2‐31:1 increased proliferation capability in vitro (**Figure** **5**A,B, Figure S5A–D, Supporting Information). To assess the co‐effects between higher lnc‐FSD2‐31:1 in tumor cells and lower ATG7 in CAFs in vivo, orthotopic xenograft models were established (Figure 5C). The tumor cells with high level of lnc‐FSD2‐31:1 expression combined with ATG7‐depleted CAFs were injected into pancreas of nude mice. In the second batch, lnc‐FSD2‐31:1‐depleted tumor cells with ATG7‐overexpressed CAFs were introduced (Figure S5E,F, Supporting Information). Our results indicated that high level of lnc‐FSD2‐31:1 in tumor cells suppressed tumor growth, and suppression of ATG7 in CAFs could rescue these effects (Figure 5D–F). IHC staining showed that the lnc‐FSD2‐31:1 level in tumor cells inhibited levels of fibronectin, collagen 1, *α*‐SMA, and Ki67, and increased levels of ATG7, whereas ATG7‐depleted CAFs would reverse these trends (Figure 5G, Figure S5G‐K, Supporting Information). In addition, other CAFs markers (vimentin and podoplanin) were also introduced to verify the status of CAFs in tumor of xenograft models. Our results showed that both vimentin and podoplanin expressed similarly in different lnc‐FSD2‐31:1 level group (Figure S5L–N, Supporting Information). Collectively, these results suggest that lnc‐FSD2‐31:1 in tumor cells induces ATG7‐dependent autophagy and suppresses fibrosis in CAFs, which contribute to antitumor efforts in PDAC.

### 2.6 MiR‐4736 Might be a Potential Therapeutic and Predictive Target of PDAC

**Figure 6:**
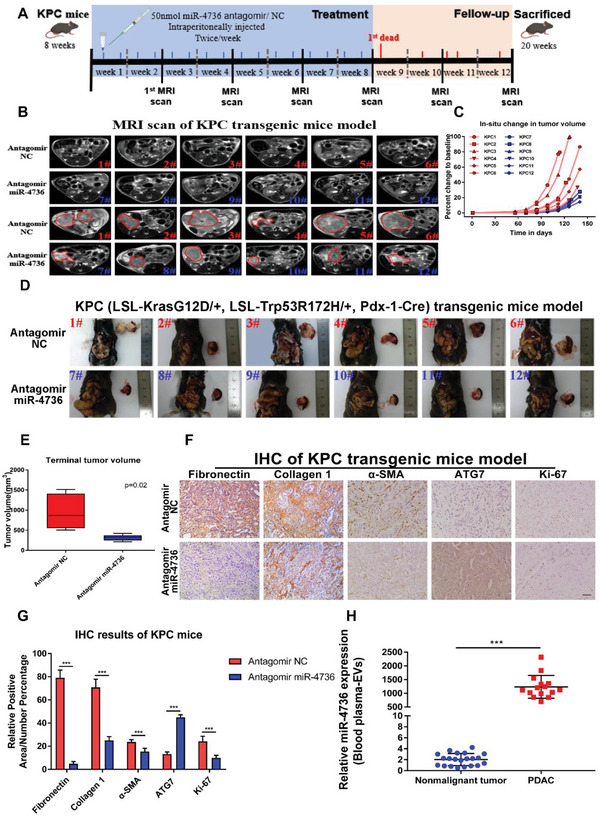
MiR‐4736 might be a potential therapeutic and predictive target of pancreatic ductal adenocarcinoma (PDAC). A) Dosing schedule for miR‐4736 antagomir in a syngeneic orthotopic tumor model. Twelve KPC (LSL‐KrasG12D/+, LSL‐Trp53R172H/+, Pdx‐1‐Cre) transgenic mice are equally divided into the miR‐4736 antagomir‐treated group and a control group, and are intraperitoneally injected with 50 nmol miR‐4736 antagomir/NC twice a week (until first death) and observed using MRI every 2 weeks. On the 58th, 66th, 71st, and 81st days after the first treatment, four control KPC mice are dead; on the 73rd day after the first treatment, one antagomir‐treated mouse dies; and on the 84th day, the remaining seven mice are sacrificed. B) Representative images of MRI scans per mouse to detect tumor changes at the initiation of treatment and before death. C) Spider plots illustrate fold changes in mean tumor volume in the treatment and follow‐up cohorts. The starting tumor volume in each mouse is assessed using MRI at Day 56. The miR‐4736 antagomir‐treated animals show stagnancy in tumor growth. D) Images show a significant decrease in harvested tumor volume in the antagomir group (*n* = 6 in each group). E) Final tumor volume in KPC mice (*n* = 6 and 6, respectively). F,G) Expression of fibronectin, collagen 1, *α*‐SMA, ATG7, and Ki67 is detected using immunohistochemistry in KPC mice (original magnification, 20×, scale bar, up 50 µm). Images are scored and the expression level is estimated as the percentage of positive area/cells using ImageJ software. H) The plasma extracellular vesicles (EVs) miR‐4736 levels in PDAC patients (*n* = 15) are higher than those in healthy donors (*n* = 21). Statistical significance is assessed using Student's *t* test. Data are shown as the mean ± SD of three replicates; ^***^ *p* < 0.001.

Due to the critical roles of miRNAs in tumor progression, the therapeutic effect of miR‐4736 in vivo was validated.^[^ ^37^ ^]^ The presence of miR‐4736 in mouse PDAC cell lines PANC‐02 and KPC (LSL‐Kras^G12D/+^, LSL‐Trp53^R172H/+^, Pdx‐1‐Cre) transgenic mouse pancreatic cancer tissue were validated (Figure S6A–C, Supporting Information). KPC mice were intraperitoneally injected with miR‐4736 antagomir or NC (50 nmol, twice a week) from week 8 and screened using MRI. We evaluated the effect of miR‐4736 manipulation based on tumor growth and tumor volume (**Figure** **6**A). The miR‐4736 antagomir‐treated group showed stagnancy in tumor growth as manifested by smaller tumor volume, decreased levels of fibronectin, collagen 1, *α*‐SMA, and Ki67, and increased levels of ATG7 (Figure 6B–G). In addition, other CAFs markers (vimentin and podoplanin) were also introduced to verify the status of CAFs in tumor of KPC mice. Our results showed that both vimentin and podoplanin expressed similarly between antagomir‐treated group and control group (Figure S6D,E, Supporting Information). These results suggest that miR‐4736 antagomir inhibits tumor progression through activating autophagy and deactivating CAFs. In addition, we isolated EVs from the plasma of 21 healthy donors and 15 PDAC patients, and the plasma EVs‐miR‐4736 level in PDAC patients was significantly higher than that in healthy donors, which suggests that miR‐4736 might be a potential therapeutic and predictive target of PDAC (Figure 6H).

## 3 Discussion

LncRNAs drive a constellation of phenotypes of cancer by acting on dysfunctional intrinsic cellular regulatory networks and intercellular communication to generate the tumor microenvironment.^[^ ^38^ ^]^ LncRNAs can bind with targeted intracellular proteins to enhance the PDAC phenotypes.^[^ ^6^ ^]^ Our previous study has demonstrated that *MALAT1* interacts with HuR, which promotes PDAC proliferation and metastasis.^[^ ^35a^ ^]^ Inversely, lncRNAs could also interact with proteins or miRNAs to inhibit the spread of complexes across different chromatin domains and affect different types of cells. For example, lncRNA *KLHDC7B* could enhance PDAC cell proliferation and macrophage M2 polarization to activate STAT3 signals in an autocrine and paracrine manner.^[^ ^39^ ^]^ In addition, lncRNAs are directly encapsulated into the EVs and transported into TME, which alter EMT and promote metastasis by activating downstream signaling pathways.^[^ ^40^ ^]^ In this study, we discovered that overexpressed lnc‐FSD2‐31:1 induced tumor suppression and ensured long‐term OS after surgery in PDAC patients. Furthermore, our study revealed that tumor‐derived lnc‐FSD2‐31:1 restrained CAFs activation via EVs‐derived miR‐4736. Based on functional prediction, we established that EVs‐derived miR‐4736 suppressed autophagy in CAFs by targeting ATG7.

KEGG pathway analysis was performed to explore the downstream signaling pathways and the underlying mechanism of lnc‐FSD2‐31:1 in PDAC progression. This analysis discovered many genetically enriched pathways, such as toxoplasmosis, osteoclast differentiation, CAMs, Th17 cell differentiation, and so on. PDAC has been distinguished from other tumors by the abundance of stroma, which consists of the extracellular matrix (ECM) and nonmalignant cells.^[^ ^41^ ^]^ The interaction of tumor cells with TME and ECM has attracted more and more attention, and we have also studied this process in the past.^[^ ^17b^ ^]^ Among the enriched pathways revealed by KEGG analysis, CAMs pathways were the most closely related to the dense stroma characteristics of PDAC; therefore, we explored the relationship between lnc‐FSD2‐31:1 level and CAFs activation, and the results proved that lnc‐FSD2‐31:1 could regulate CAFs via EVs miR‐4736‐ATG7 axis.

In our study, *α*‐SMA was used as a marker for CAFs to show that they were activated by lnc‐FSD2‐31:1‐EVs miR‐4736‐ATG7 axis in PDAC. Traditionally, CAFs are defined as “*α*‐SMA positive” nonmalignant cells, and considered as tumorigenic cells.^[^ ^41^, ^42^ ^]^ However, targeting therapies that inhibit CAFs have failed in PDAC,^[^ ^43^ ^]^ suggesting that CAFs may be more complex than it was initially thought.^[^ ^44^ ^]^ At an early stage of PDAC, evidences on the existence of different subtypes of CAFs are emerging.^[^ ^45^ ^]^ Afterward, Öhlund et al. established a three‐dimensional organotypic coculture in vitro to demonstrate that the formation of myofibroblastic CAFs (myCAFs) is contact‐dependent, whereas inflammatory CAFs (iCAFs) may be enriched in the absence of contact with PDAC cells, and they convert between these types in a dynamic fashion.^[^ ^17c^ ^]^ They also proved that myCAFs are located in the periglandular region, whereas iCAFs are located more distantly from neoplastic cells and myCAFs in vivo. A. Hosein et al. used single‐cell RNA sequencing to demonstrate the existence of three distinct molecular subtypes of fibroblasts in the normal mouse pancreas, which ultimately gave rise to two distinct populations of fibroblasts in advanced PDAC.^[^ ^46^ ^]^ Coincidently, Elyada et al. also used single‐cell RNA sequencing to characterize myCAFs and iCAFs in human PDAC, but they proved that iCAFs, myCAFs, and antigen‐presenting CAFs (apCAFs) existed in KPC mouse model of PDAC.^[^ ^27^ ^]^ Furthermore, they found that apCAFs were predominantly admixed with the iCAF, and the image mass cytometry and multiple antibodies were used to identify apCAFs in human PDAC.^[^ ^27^ ^]^ At last, they observed that apCAFs formed a dynamic fibroblast population and that they may require environmental cues to be maintained as a subpopulation. Collectively, studies on CAFs heterogeneity are still in their descriptive stage through single‐cell sequencing. However, there is no consensus on the classification criteria and CAF markers for heterogeneity. Furthermore, according to the different location and environment stimulation, CAF subtype state is undergoing dynamic transformation. Nevertheless, the reasons for CAF heterogeneity, the mechanisms that control dynamic transformation of CAFs, and specific roles of CAF subtypes remain unclear. In our study, in addition to *α*‐SMA that was used as the marker in coculture experiments, we also used vimentin and podoplanin to do the recognition assays (vimentin and podoplanin have been proved expressed in both CAF subpopulations^[^ ^27^ ^]^). We found that tumor cells‐derived lnc‐FSD2‐31:1 restrained myCAFs activation, but this was not the case for all CAF subtypes. Our study indirectly demonstrated the existence of CAFs heterogeneity in PDAC, and myCAFs were regulated by lnc‐FSD2‐31:1‐EVs‐miR‐4736‐ATG7 axis in PDAC. Recently, the Banbury Center meeting discussed CAFs biology and issued a consensus statement; they pointed out the subspecialization of CAFs and switching between distinct functional states. Transgenic manipulations and coinjection assays are the main methods to explore CAFs functions in vivo.^[^ ^41b^ ^]^ Once the tumor model is established, various metrics relating to CAFs function should be measured, including matrix organization and cross‐linking, tissue mechanics, tumor vascularization, tumor growth, metastatic spread, immune infiltrate, and therapy response. However, the consensus suggests that this approach is cost and time consuming; thus, posing a barrier for many researchers.^[^ ^41b^ ^]^ Therefore, the objective construction of CAFs research model, as well as the accurate and effective methods to study the heterogeneity of CAFs need to be investigated. Biffi, G. et al. showed that the TGF‐*β*/SMAD2/3 pathways induced myCAFs activation.^[^ ^47^ ^]^ CXCL3‐CXCR2 signaling has been shown to convert CAFs into myCAFs, targeting adhesive molecule‐type III collagen and inducing PDAC metastasis.^[^ ^48^ ^]^ In addition, Sonic Hedgehog signaling is preferentially activated in myCAFs, and Hedgehog pathway antagonist, LDE225, inhibits tumor growth by reducing myCAFs abundance.^[^ ^17^, ^49^ ^]^ Inversely, several studies reported that myCAFs had tumor restraining activity.^[^ ^43^, ^44^, ^49^, ^50^ ^]^ Our study confirmed that lnc‐FSD2‐31:1 in tumor cells acted as the suppressor of tumor progression; thus, we infer that myCAFs have tumor promoting activity in our experiments. As the crucial nonmalignant component of TME, CAFs have been studied by investigating tumor cells derived noncoding RNA signals.^[^ ^50b^ ^]^ As more effects of noncoding RNAs are being discovered, the mechanisms of action of tumor cells residing in heterogeneous TME will be elucidated.

Inhibition of CAFs presents failed antitumor roles in PDAC,^[^ ^17b^ ^]^ remaining that CAFs may play dual roles in tumor progression. Our study revealed that EVs‐derived miR‐4736 ameliorated CAFs autophagy and improved fibrotic phenotypes. These results indicate that CAFs are heterogeneous under signal alterations in TME. Autophagy is believed to be positively correlated with the tumor occurrence and metastasis.^[^ ^51^ ^]^ Autophagy has protumorigenic roles through promoting immune evasion in PDAC by degrading MHC‐I,^[^ ^52^ ^]^ or suppressing antitumor immunity mediated by CD8^+^T cells.^[^ ^53^ ^]^ Conversely, autophagy also achieves antitumorigenic effects, including maintenance of genomic stability, suppression of oxidative stress, and inhibition of NRF2 activation.^[^ ^36^ ^]^ Under the KEGG enrichment analysis, we found and proved that ATG7 was the downstream target of lnc‐FSD2‐31:1 in vivo and in vitro. In our previous study, we have explored the autophagy in PDAC.^[^ ^35^ ^]^ With regard to the enrichment of stromal cells in pancreatic tumors, the autophagy of CAFs attracted our attention. Currently, there are studies underlining autophagy of CAFs in PDAC, whereas only few explore autophagy in pancreatic stellate cells (PSCs) and in PDAC development. PSC has been demonstrated to support tumor metabolism by autophagic alanine secretion. Alanine secreted by PSCs was dependent on autophagy, which competed for glucose and glutamine‐derived carbon in PDAC to fuel the tricarboxylic acid (TCA) cycle.^[^ ^54^ ^]^ Endo et al. found that activated autophagy in PSCs produced ECM molecules and IL‐6 and was associated with shorter survival and recurrence in PDAC patient. Inhibition of ATG7 in PSCs decrease the fibrosis and proliferation. In our study, autophagic activation of CAFs was negatively correlated with tumor growth and fibrotic progression in PDAC, demonstrating that activated autophagy restrained CAFs activation and reduced fibrosis. Our study showed the opposite results with previous research. It may be due to different activation states between PSCs and CAFs. However, the intrinsic cross‐talks between tumor cells and CAFs could not be ignored. Consequently, further evidences may explain the complexed roles of autophagy in CAFs and PDAC development.

MiRNA antagomir, characterized by its conserved structure and broad effects in human disease, is regarded as a precise and effective targeted therapy.^[^ ^26^, ^55^ ^]^ In our study, injection of miR‐4736 antagomir showed potential treatment strategy for PDAC in KPC mice. Currently, three miRNA mimic or anti‐miRNA clinical trials (NCT02826525, NCT03601052, and NCT0120042) are ongoing. The targeted therapy of miRNA may bring hope for PDAC treatment. Nevertheless, EVs are presented as biomedical drug capsule, accompanied with higher stability, pharmacokinetics, better biocompatibility, and targetability.^[^ ^56^ ^]^ EVs can be easily detected in body fluids, and EV‐derived miRNAs can be used as indicators for predicting OS^[^ ^57^ ^]^ and metastasis.^[^ ^58^ ^]^ A dual‐SERS biosensor has been developed to detect early PDAC by one‐step text of exosome miRNA in blood.^[^ ^59^ ^]^ In our study, miR‐4736 was significantly elevated in EVs of blood plasma collected from PDAC patients, which suggests that it could serve as a potential biomarker for PDAC early diagnosis.

We recognize potential limitations of current study. First, apart from Lnc‐FSD2‐31:1, a bulk number of lncRNAs upregulated in the long‐term survival patients may also contribute to CAFs activation. Systematical analysis of all possible targets might be a quest difficult to full‐fill in current study. Second, lnc‐FSD2‐31:1 was demonstrated to influence tumor growth independently, and downstream pathways were predicted by KEGG analysis. Due to difficulty of using systems biology research methods in this project, the missing part of mechanisms will be further discussed. Ultimately, the instability of CAFs state and limited techniques restrict us exploring the heterogeneity of CAFs accurately. Further studies are expected to better understand the roles of CAFs subtypes in PDAC progression.

**Figure 7:**
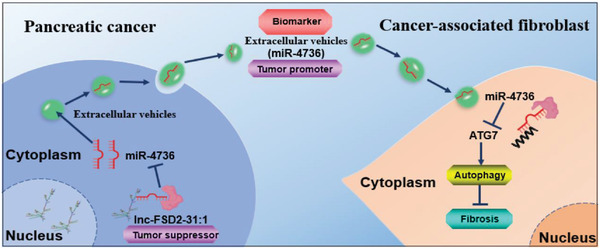
Schematic diagram. Tumor cells‐derived lnc‐FSD2‐31:1 restrains cancer‐associated fibroblasts (CAFs) activation via changing extracellular vesicles (EVs) derived miR‐4736 amount in vitro and in vivo. EVs‐derived miR‐4736 targeted ATG7 in CAFs, which suppressed autophagy and contributed to CAFs activation.

In summary, we discovered that tumor‐derived lnc‐FSD2‐31:1 regulated autophagy and fibrosis of CAFs via EVs cargo miR‐4736. MiR‐4736 may provide an underlying target for PDAC diagnosis and treatment (**Figure** **7**).

## 4 Experimental Section

### Patients and Tissues

Tissues (102 PDAC and 40 adjacent normal) from PDAC patients who underwent pancreatic resection in the Department of Pancreatic and Biliary (The First Affiliated Hospital of Harbin Medical University, Harbin, Heilongjiang, China) from January 2013 to June 2021. The experiments were approved by the Ethics Committee of the First Affiliated Hospital of Harbin Medical University (IRB‐AF/SC‐04/02.0).

### Cell Lines and Culture

Human pancreatic duct epithelial (HPDE) cells were purchased from ATCC. BxPC‐3, PANC‐1, and PANC‐02 cells were purchased from the Type Culture Collection of the Chinese Academy of Sciences (Shanghai, China). Cells were used within 6 months after ordering and cultured in DMEM (Gibco, USA), RPMI 1640 (HyClone, USA), 10% FBS (Gibco, USA), and 1% penicillin/streptomycin with 5% CO2 at 37 °C.

### Transfection

Transfection was performed as described previously.^[^ ^42^ ^]^ Full‐length *lnc‐FSD2‐31:1* and ATG7 were synthesized and subcloned into a lentiviral expression vector. Lentiviruses with scrambled shRNA against *lnc‐FSD2‐31:1* and ATG7 were constructed (GeneChem, Shanghai China). Both the control vector and negative control were transfected into cells to produce lentivirus, which was selected by 2.5 µg mL^−1^ puromycin (Sigma, USA) for 2 weeks. The target sequences of the lentiviruses are listed in Supporting Information. MiR‐4736 mimic, miR‐4736 inhibitor, and their negative control were designed and synthesized by RiboBio (RiboBio, Guangdong, China). They were transiently transfected into cells by Lipofectamine 2000 (Life Technologies Limited Paisley, Grand Island, NY, USA).

### Western Blot Analysis

Western blot analysis was performed as described previously.^[^ ^42^ ^]^ Proteins were extracted from cells, EVs, and immunoprecipitated using RIPA buffer (Beyotime, China). Total protein (20‐40 µg) was subjected to 8%, 10%, and 12% SDS–PAGE and transferred to nitrocellulose membranes. The membranes were blocked with 5% skim milk and incubated with antibodies overnight at 4 °C. The second day, the membranes were washed and incubated with secondary antibodies (Santa Cruz Biotech, USA). The bands were visualized with the Molecular Imager System (BIO‐RAD, USA). The antibodies are listed in Supporting Information.

### RNA Isolation, Reverse Transcription, and Quantitative Real‐Time PCR

Total RNA from cells and tissues was extracted using the AxyPrep Multisource Total RNA Miniprep Kit from Axygen (Corning, Jiangsu, China). First‐strand cDNA was synthesized by Rever TraAce qPCR RT Master Mix with gDNA Remover Kit (FSQ‐301, FSQ‐101, TOYOBO, Japan). qRT‐PCR was performed as previously described.^[^ ^42^ ^]^ qRT–PCR (SYBR Green Assay, Roche Diagnostics GmbH, USA) was conducted on a 7500 FAST Real‐Time PCR System (Applied Biosystems). The relative expression levels of lncRNAs, miRNAs and mRNAs were calculated using the 2−ΔΔT method. *GAPDH* and *U6* were used as endogenous controls. Some of primer sequences are listed in Supporting Information and others were purchased from Comate Bioscience (Institute of Biotechnology, Jilin, China) and RIBOBIO (Guangdong, China).

### CRISPR/Cas9 System

GENECHEM designed and cloned the U6‐sgRNA(lnc‐FSD2‐31:1) *4 into CV279 plasmid. Lentiviruses were produced using KpnI/NheI, and the element sequence was MCS‐EF1a‐Cas9‐FLAG‐P2A‐puro (http://www.genechem.com.cn/index/supports/tool_search.html?keywords = CV279). The sgRNA sequences were: sgRNA1, 5’‐CGCGCTGGGGACGTTATGGT‐3’, sgRNA2, 5’‐ATATTCTTGAGATCGGTACA‐3’. sgRNA3, 5’‐AAGCAGAATACTACAACTCC‐3’. sgRNA4, 5’‐TAAAAAGGCACTACTAGATT‐3’. The verification method used was the nonmismatch enzyme method and PCR amplification (U6‐sgRNA(lnc‐FSD2‐31:1) *4, outer primers, and inner primers were listed in Supporting Information). PANC‐01 cells were infected with the complete medium containing corresponding virus, after 48 h infection, the puromycin selection (2.0 µg mL^−1^) was initiated and single cell cloning technique was used to isolate KO cells. Heterodimerization and digestion were performed with the Knockout and Mutation Detection Kit (GENECHEM) according to the manufacturer's instructions. Cleavage products were separated on a 2% agarose gel and stained with ethidium bromide. Images were captured with the SmartGel Image Analysis System. The knockout efficiency of cells was tested by qRT–PCR.

### EdU (5‐Ethynyl‐2’‐Deoxyuridine) Assay

Cell proliferation experiments were performed using an EdU assay kit (C10310‐1/2/3, RiboBio). In summary, the cells were treated with 10 mmol L^−1^ EdU for 2 h at 37 °C, fixed with 4% paraformaldehyde for 30 min, incubated with 2 mg mL^−1^ glycine for 5 min, washed with PBS, and treated with 0.5% Triton X‐100 for 10 min. Then, 100 µL of 1X Apollo was added, and the cells were washed with PBS again. Next, the cells were observed in a fluorescence microscopy (20×, Nikon, Japan).

### Cell Counting Kit‐8 (CCK‐8) Assay

The cells from different groups were distributed into 96‐well plates (100 µL well^−1^; 3000 cells well^−1^) and incubated at 37 °C with 5% CO2. At 12, 24, 36, 48, and 60 h of incubation, CCK‐8 solution (10 µL well^−1^) was incubated with each well for 2 h. Then the absorbance was measured at 450 nm (ELx808, BioTek, USA).

### Fluorescence In Situ Hybridization

FISH was performed as previously described.^[^ ^35a^ ^]^ FISH was performed with the Fluorescent In Situ Hybridization Kit (RiboBio). The sections were fixed in 4% formaldehyde for 10 min, permeabilized in PBS with 0.5% Triton X‐100 at 4 °C for 5 min, washed with PBS three times for 5 min, and prehybridized at 37 °C for 30 min. Then, the hybridization probes of lnc‐FSD2‐31:1 (RiboBio), U6 (RiboBio), and 18S (RiboBio) were used in the hybridization solution at 37 °C overnight. The second day, the sections were washed and counterstained with DAPI and imaged in a fluorescence microscope (×20, Nikon, Japan).

### Extracellular Vesicles Isolation and Identification

A total of 1–2 × 10^6^ PDAC cells were cultured in 10 cm plates with 6.5 mL normal media. When cell confluence was about 60–70%, the original media was removed and replaced with EVs‐depleted media (EVs removed using ultracentrifugation, 110 000 × *g* for 18 h) for 48 h. When cell confluence reached about 90–95% without suspended cells, the supernatant (50 mL from eight plates) was collected for extraction of EVs. The cell debris was discarded after culture medium centrifugation at 300 × *g*, 2000 g, and 10 000 × g, and then the supernatant was centrifuged at 110 000 × *g* for 70 min (all steps were performed at 4 °C). The pellet containing the EVs was collected and resuspended in PBS (0.1 mL). EVs were observed by transmission electron microscopy (TEM) (JEOL 1200EX, Japan) and quantified using Western blot (the EVs characteristic proteins Alix, CD63, and TS101) and Nano‐Sight NS300 (Malvern Instruments Ltd, UK). Plasma EVs isolated from the 21 healthy donors and 15 PDAC patients, the experiments were approved by the Ethics Committee of the First Affiliated Hospital of Harbin Medical University (IRB‐AF/SC‐04/02.0).

### Transmission Electron Microscopy

CAFs were fixed in 2.5% glutaraldehyde, washed three times with 0.1 m orthophosphoric acid, postfixed in 1% osmium tetroxide buffer, washed three times again with 0.1 m orthophosphoric acid, dehydrated with ethanol and acetone, embedded in spur resin, and cut into thin sections. The sections were stained with a saturated solution of uranyl acetate and lead citrate. Sections were examined at 80 kV using a JEOL 1200EX TEM.

### Immunofluorescence

IF was performed as previously described.^[^ ^42^ ^]^ The sections (tissues or cells) were fixed with 4% paraformaldehyde for 30 min, permeabilized with 0.5% Triton X‐100 for 20 min, and then incubated with antibodies for 2 h, washed with PBS three times, and incubated with secondary antibodies for 1 h. Finally, the sections were counterstained with DAPI and detected using fluorescence microscopy (×20, Nikon, Japan). The antibodies are listed in Supporting Information.

### Isolation and Primary Culture of CAFs

Fresh human PDAC tissues were collected and minced and digested with enzymes (0.1 mg mL^−1^ hyaluronidase, 0.1 mg mL^−1^ DNase, and 2 mg mL^−1^ collagenase). After filtration and centrifugation, the cells were plated on poly‐l‐lysine‐coated plates in DMEM/F‐12 and HEPES (Gibco, USA) with 20% FBS (Gibco, USA). The remaining minced tissues were cultured in DMEM/F‐12 and HEPES (Gibco, USA) with 20% FBS (Gibco, USA) directly. Cells were screened by differential adherence and identified using IF and flow cytometry.

### Flow Cytometry Analysis

The primary CAFs were digested with 0.25% trypsin, blocked with 10% FBS, incubated with anti‐Fibroblast activation protein *α* (FAP) antibodies (Invitrogen, USA) for 1 h, washed with PBS, incubated with IC Fixation Buffer (eBioscience, USA) for 1 h, washed with PBS, incubated with anti‐*α*‐SMA antibodies (Abcam, Cambridge, UK) for 1 h, washed with PBS, incubated with mouse anti‐rabbit IgM/APC antibody (Bioss, Beijing) and rabbit anti‐mouse IgG‐Fc/PE antibody (Bioss, Beijing) for 1 h, washed with PBS, and analyzed with a flow cytometer (NovoCyte 3110, Agilent, USA).

### Immunohistochemical Staining

The immunohistochemical staining protocol has been described previously.^[^ ^41b^ ^]^ Specimens were fixed in 10% buffered formalin for 24 h, embedded in paraffin, sectioned (5 mm). The sections were deparaffinized and rehydrated, followed by incubation in 3% hydrogen peroxide for 15 min, rinsing in water and blocking in 2.5% normal horse serum. The sections were incubated with antibodies and captured by microscopy (20×, Nikon, Japan). The antibodies are listed in Supporting Information. The total number of positive pixels was counted in five fields and calculated using ImageJ.

### RNA Immunoprecipitation

RIP was performed using a RIP Kit (Bes5101, BersinBio, China) according to the manufacturer's protocols. In summary, CAFs were collected and lysed in lysis buffer and then incubated with magnetic beads coated with anti‐AGO2 (Abcam), anti‐ATG7 (Cell Signaling Technology), or anti‐IgG antibodies. Afterward, the immunoprecipitated RNAs were extracted as described above. The coprecipitated RNAs were then detected using qRT–PCR.

### Dual Luciferase Reporter Assay

The wild‐type and two mutated binding sites of ATG7 were cloned into the pGL3.10 vector and cotransfected with miR‐4736 NC or miR‐4736 mimic into PANC‐02 cells. After 48 h, the luciferase activity was detected using a Dual‐Luciferase Reporter Assay System (Promega) and normalized using Renilla luciferase activity. The pGL3.10 plasmids were designed and purchased from GenScript (Nanjing, China). The sequences are listed in the Supporting Information.

### Orthotopic Xenograft Models

The experimental protocol was approved by the Institutional Review Board of the First Affiliated Hospital of Harbin Medical University (IRB‐AF/SC‐04/02.0). Six‐week‐old female BALB/c nude mice were purchased from Charles River Company (Beijing). PANC‐1 cells expressed different levels of lnc‐FSD2‐31:1 and CAFs expressed different levels of ATG7 by lentiviral transfection and mixed at a 3:1 ratio. Mice were anesthetized with intraperitoneal injections of 2.5% 2,2,2‐tribromoethanol (Sigma–Aldrich, Shanghai), and mixed cells (106 cells per mouse) were orthotopically injected into the pancreas of nude mice. The mice were imaged weekly using the NightOWL II LB983 In vivo imaging system (BERTHOLD TECHNOLOGIES GmbH & Co. KG, Germany). After 5 weeks, all mice were euthanized, the weight of tumors and the number of abdominal metastases were recorded, and all the specimens were fixed in 4% formaldehyde.

### Treatment of KPC Transgenic Mice

Eight‐week‐old C57BL/6‐Trp53em4(R172H)Krasem4(LSL‐G12D)Tg(Pdx1‐cre)Smoc male mice were purchased from Model Organisms (Shanghai). Mice were intraperitoneally injected with 50 nmol miR‐4736 antagomir/NC (RiboBio) twice a week (until first death), and the mice were observed using MRI every 2 weeks. Except for the dead mice, the remaining mice were euthanized after 84 days of treatment. The volume of tumors was recorded and fixed in 4% formaldehyde.

### Statistical Analysis

Prism 7.04 (Graph Pad) and ImageJ software were used to perform statistical and pictural analyses. Data are expressed as the mean ± standard deviation. Kaplan–Meier survival analysis was used to evaluate overall survival. ANOVA, Student's *t*‐test, and Spearman correlation were used to evaluate statistical significance and power analysis. Data are shown as the mean ± SD of three replicates. Differences were considered significant when *p* < 0.05.

## Authors’ Contributions

H.Z.C. and B.S. contributed equally to this work as co‐corresponding authors. X.L.G. and L.L. were co‐first authors. X.L.G., L.L., H.Z.C., and B.S. conceptualized the study. X.L.G., L.L., Y. L, W.B.Y., J.S.H., Z.J.Z., C.D.C., T.Z., H.Z.C., and B.S. developed methods. X.L.G., L.L., Y. L, W.B.Y., Y.Y.Z., L.W.L., Y.X., G.Q.L., D.X.L., R.B., X.W.B., G.W., H.C., Y.W.W., H.Z.C., and B.S. performed experiments. X.L.G., L.L., and H.Z.C. analyzed data. X.L.G., L.L., and H.Z.C. wrote the manuscript. All authors revised the manuscript and approved the final version. H.Z.C. and B.S. acquired funding and supervised.

## Conflict of Interest

The authors declare no conflict of interest.

## Ethics Approval and Consent to Participate

PDAC and normal pancreatic tissues were acquired from patients undergoing a surgical procedure at The First Affiliated Hospital of Harbin Medical University (Harbin, China) (IRB‐AF/SC‐04/02.0). Patient consent was obtained prior to the initiation of the study.

## Supporting information

Supporting Information

## Data Availability

The data that support the findings of this study are openly available in Hongze Chen at ht tps://doi.org/[0000‐0002‐4805‐4150], reference number 1.
