# Supplementary material for: Tumor Cell Derived Lnc‐FSD2‐31:1 Contributes to Cancer‐Associated Fibroblasts Activation in Pancreatic Ductal Adenocarcinoma Progression through Extracellular Vesicles Cargo MiR‐4736
Source: Adv Sci (Weinh). 2023 Feb 2;10(10):2203324. doi: 10.1002/advs.202203324 (PMC10074102; doi:10.1002/advs.202203324)
Supplement: Supplementary file 1 — Supporting Information [file ADVS-10-2203324-s001.pdf]

Supporting Information

Tumor Cell Derived Lnc-FSD2-31:1 Contributes to Cancer-Associated Fibroblasts Activation in Pancreatic Ductal Adenocarcinoma Progression through Extracellular Vesicles Cargo MiR-4736

Xinglong Geng, Le Li, Yan Luo, Wenbo Yang, Jisheng Hu, Zhongjie Zhao, Chundong Cheng, Tao Zhang, Yangyang Zhang, Liwei Liu, Yu Xie, Guanqun Li, Danxi Liu, Rui Bai, Xuewei Bai, Gang Wang, Hua Chen, Yongwei Wang, Hongze Chen, and Bei Sun**

X. Geng, L. Li, Y. Luo, W. Yang, J. Hu, Z. Zhao, C. Cheng, T. Zhang, Y. Zhang, L. Liu, Y. Xie, G. Li, D. Liu, R Bai, X. Bai, G. Wang, H. Chen, Y. Wang, H. Chen, B. Sun
Department of Pancreatic and Biliary Surgery, The First Affiliated Hospital of Harbin Medical University
Key Laboratory of Hepatosplenic Surgery, Ministry of Education, The First Affiliated Hospital of Harbin Medical University
Harbin, Heilongjiang 150000, China

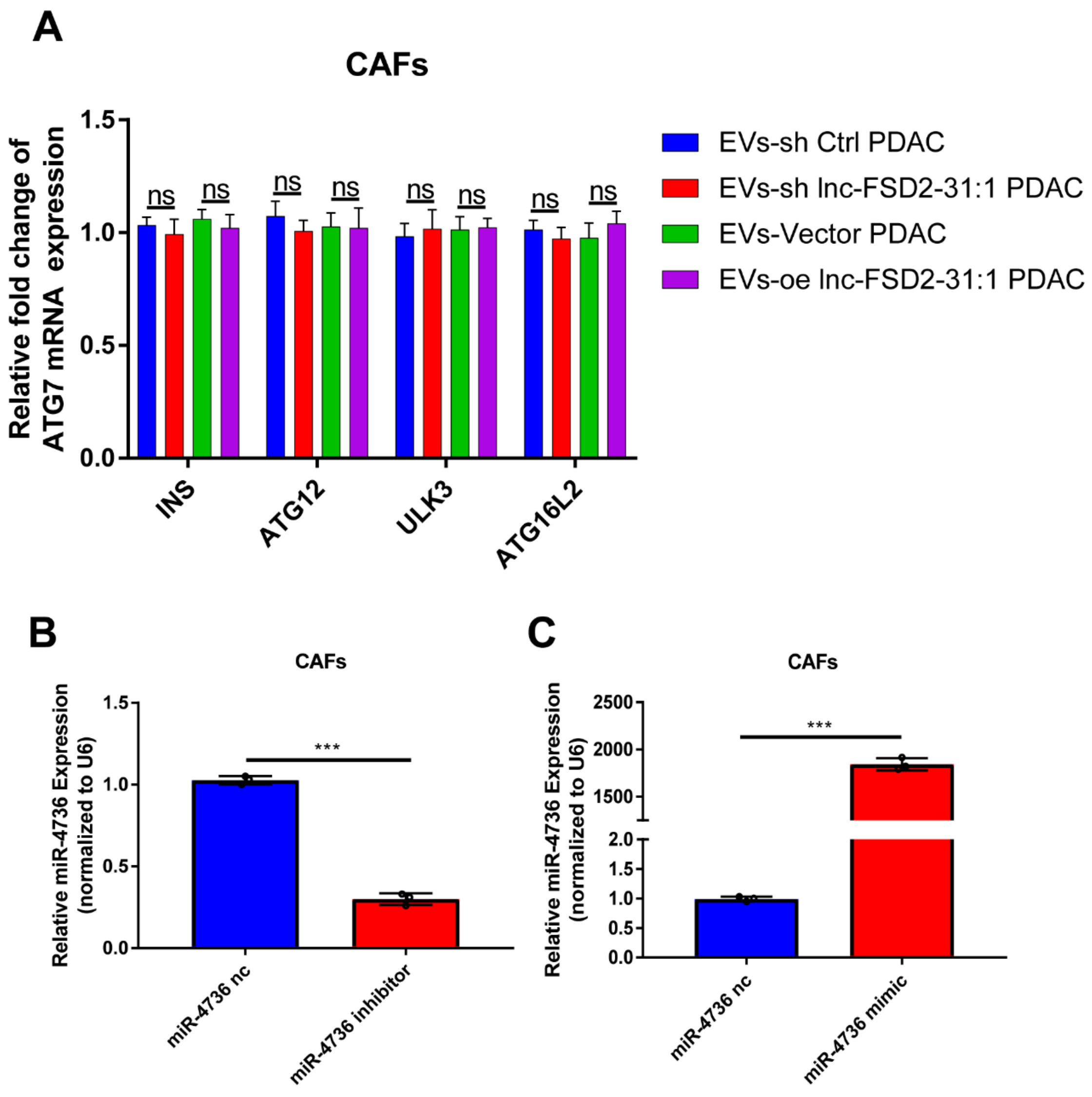

Figure S1. Overexpression of lnc-FSD2-31:1 predicts long-term survival in PDAC. A) Volcano map of lncRNAs expression between long-term and short-term survival PDAC patient tissues. (n=3, long-term >5 years, short-term <6 months). A total of 489 lncRNAs are identified, different RNAs are screened according to the criteria of log2FC > 2 and *p*-value < 0.05. Duplicate RNAs, coding genes (https://lncipedia.org/), and genes less than 200 bp are filtered out. The studied lncRNAs are excluded. After the initial screening, the remaining lncRNAs are sequenced according absolute foldchange. Finally, the "top 10" discrepant lncRNA expressions are tested in 89 PDAC and 40 cancer adjacent tissues using qRT-PCR. B) qRT–PCR assays detecte 9 lncRNAs (9 of the top 10) in 89 PDAC and 40 cancer adjacent tissues. C-D) The noncoding nature of lnc-FSD2-31:1 is confirmed by ORF Finder and codon substitution frequency scores. Statistical significance is assessed using Student's *t*-test. Data are shown as the mean ± SD of three replicates; ns: not significant.

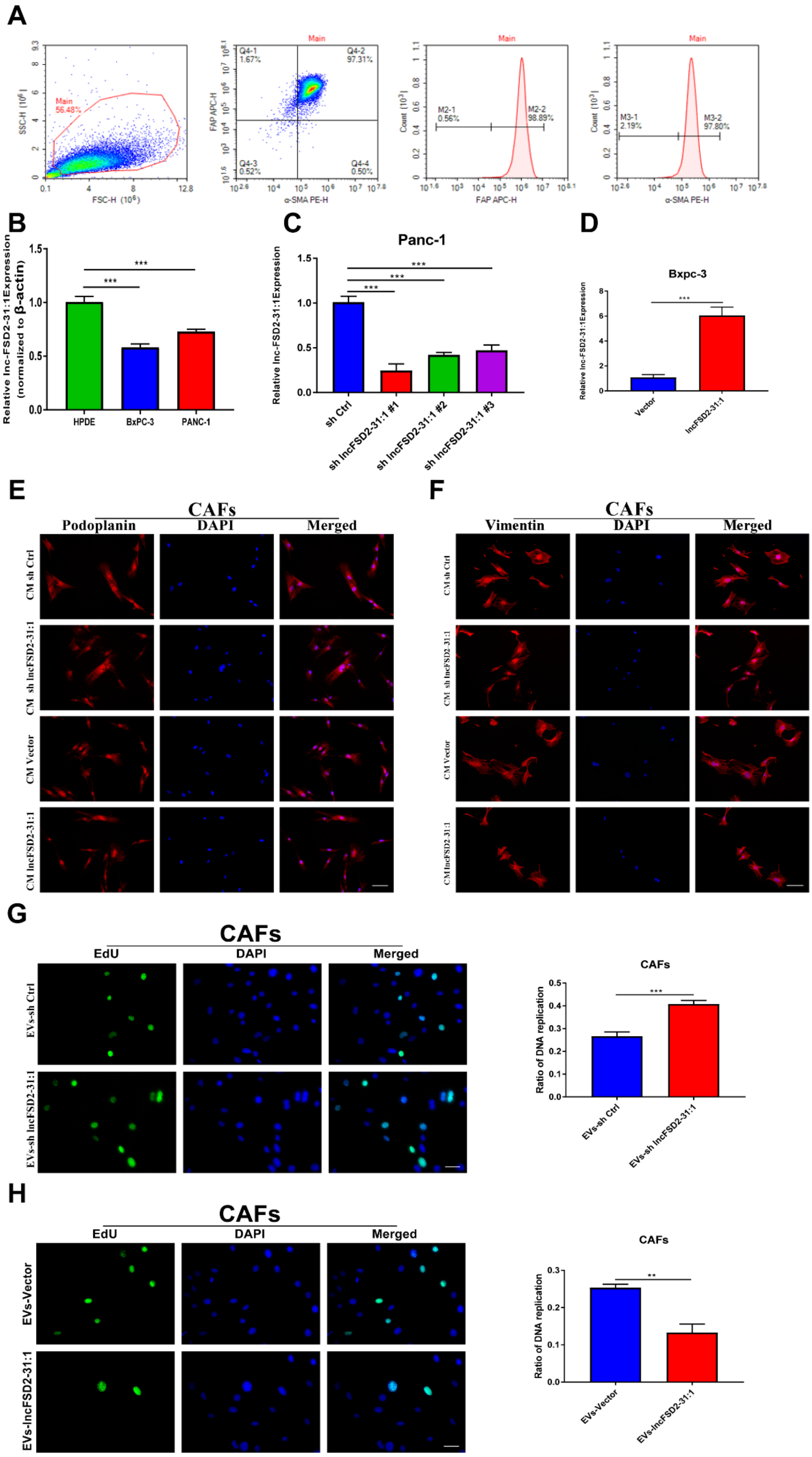

A
Main 56.48%
SSC-H (10^6)
FSC-H (10^6)
Q4-1 1.67%
Q4-2 97.31%
Q4-3 0.52%
Q4-4 0.50%
FAP APC-H
α-SMA PE-H
M2-1 0.56%
M2-2 98.89%
M3-1 2.19%
M3-2 97.80%
Count (10^3)
B
Relative lnc-FSD2-31:1Expression (normalized to β-actin)
HPDE
BxPC-3
PANC-1
C
Panc-1
Relative lncFSD2-31:1Expression
sh Ctrl
sh lncFSD2-31:1 #1
sh lncFSD2-31:1 #2
sh lncFSD2-31:1 #3
D
Bxpc-3
Relative lnc-FSD2-31:1Expression
Vector
lncFSD2-31:1
E
CAFs
Podoplanin
DAPI
Merged
CM sh Ctrl
CM sh lncFSD2-31:1
CM Vector
CM lncFSD2-31:1
F
CAFs
Vimentin
DAPI
Merged
G
CAFs
EdU
DAPI
Merged
EVs-sh Ctrl
EVs-sh lncFSD2-31:1
Ratio of DNA replication
H
CAFs
EVs-Vector
EVs-lncFSD2-31:1

Figure S2. Tumor-derived lnc-FSD2-31:1 restrains CAFs activation through extracellular vesicles. A) Flow cytometry results show that 97.31% of the isolated CAFs are simultaneously positive for fibroblast activation protein α (FAP) and α-SMA. B) The expression of lncRNAs in human pancreatic ductal epithelial (HPDE) cells, PANC-1 cells, and BxPC-3 cells is detected using qRT–PCR assays. C) Lnc-FSD2-31:1 is effectively silenced in PANC-1 cells through lentivirus transfection. D) Lnc-FSD2-31:1 was effectively overexpressed in BxPC-3 cells through lentivirus transfection. E-F) Immunofluorescence detection of podoplanin and vimentin expression in CAFs cultured with CM from PANC-1 cells with different lnc-FSD2-31:1 expressions level (original magnification, 20×, scale bar, 50 μm). G-H) Proliferative viability is determined via EdU retention assays in CAFs cocultured with EVs from pancreatic cancer with different lnc-FSD2-31:1 expression level (Original magnification, 20×, bar, 50 μm). Statistical significance was assessed by Student's *t* test. Data are shown as the mean ± SD of three replicates; $^{**}P < 0.01$; $^{***}P < 0.001$.

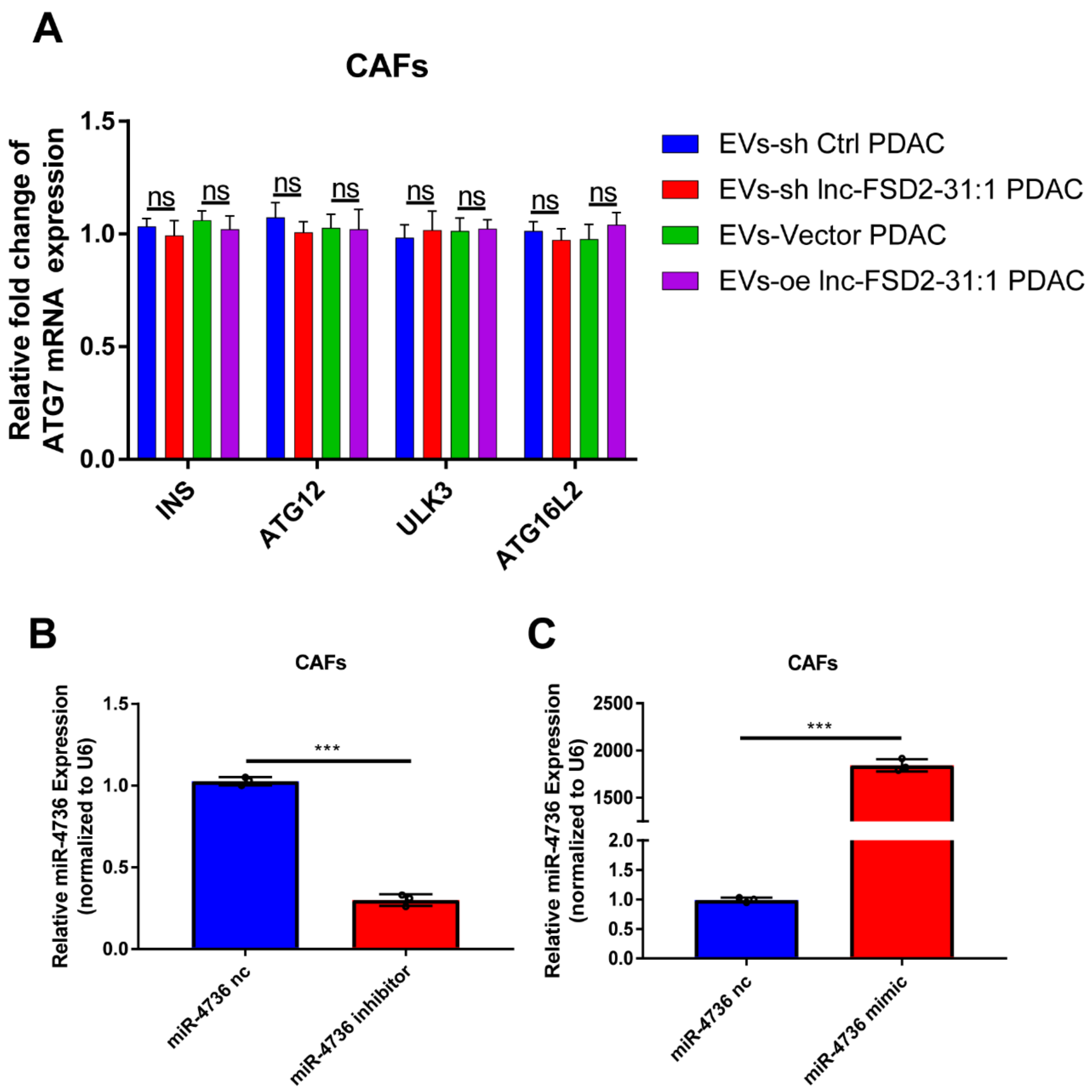

Figure S3. Cancer cells-derived EVs restrains CAFs activation via lnc-FSD2-31:1-miR-4736-ATG7 axis. A) CAFs cocultured with EVs derived from lnc-FSD2-31:1 -high/-low tumor cells and RNAseq is performed, 4 of 5 (except for *ATG7*) discrepant mRNAs enriched in autophagy pathway are verified to be no statistically significant. B-C) Transfection with miR-4736 mimic/inhibitor effectively increases/decreases miR-4736 expression in CAFs. Statistical significance was assessed by Student's *t*-test. Data are shown as the mean ± SD of three replicates; ***$P < 0.001$.

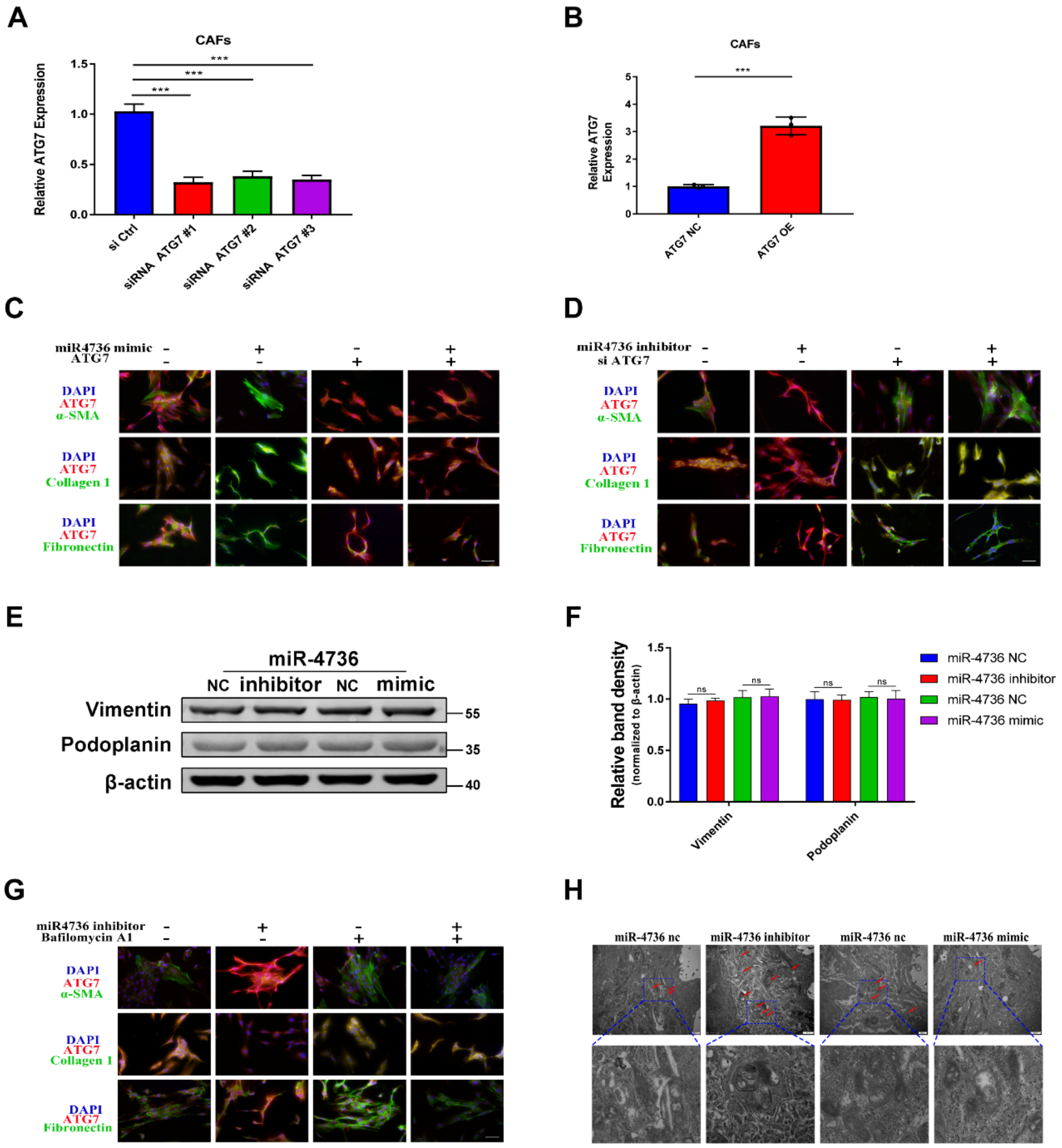

Figure S4. MiR-4736 targets ATG7 and restrains autophagy in CAFs. A-B) *ATG7* mRNA is effectively silenced and overexpressed in CAFs through siRNA against the ATG7/ATG7 overexpression plasmid. C-D) Observing the dynamic changes in fibrosis and autophagy using immunofluorescence in CAFs, α-SMA, collagen 1, fibronectin (green), and ATG7 (red). The cohort is the same as Figure 4 E and G (original magnification, 20×, scale bar, 50 μm). E-F) After being cultured with miR-4736 inhibitor or mimic, CAFs are subjected to western blotting to test vimentin and podoplanin expression. The relative band densities are calculated and there is no statistically significant difference in intensity between them. G) Immunofluorescence assays show that miR-4736 inhibitor can decreased fibrosis levels and increase

autophagic flux in CAFs, while Bafilomycin A1 simultaneously blocks these effects in CAFs. H) TEM shows the different counts of autophagosomes in CAFs transiently transfected with miR-4736 inhibitor/mimic. The red arrow shows autophagosomes (scale bar, 100 nm). Statistical significance is assessed using Student's *t*-test. Data are shown as the mean ± SD of three replicates; ***P < 0.001; ns: not significant.

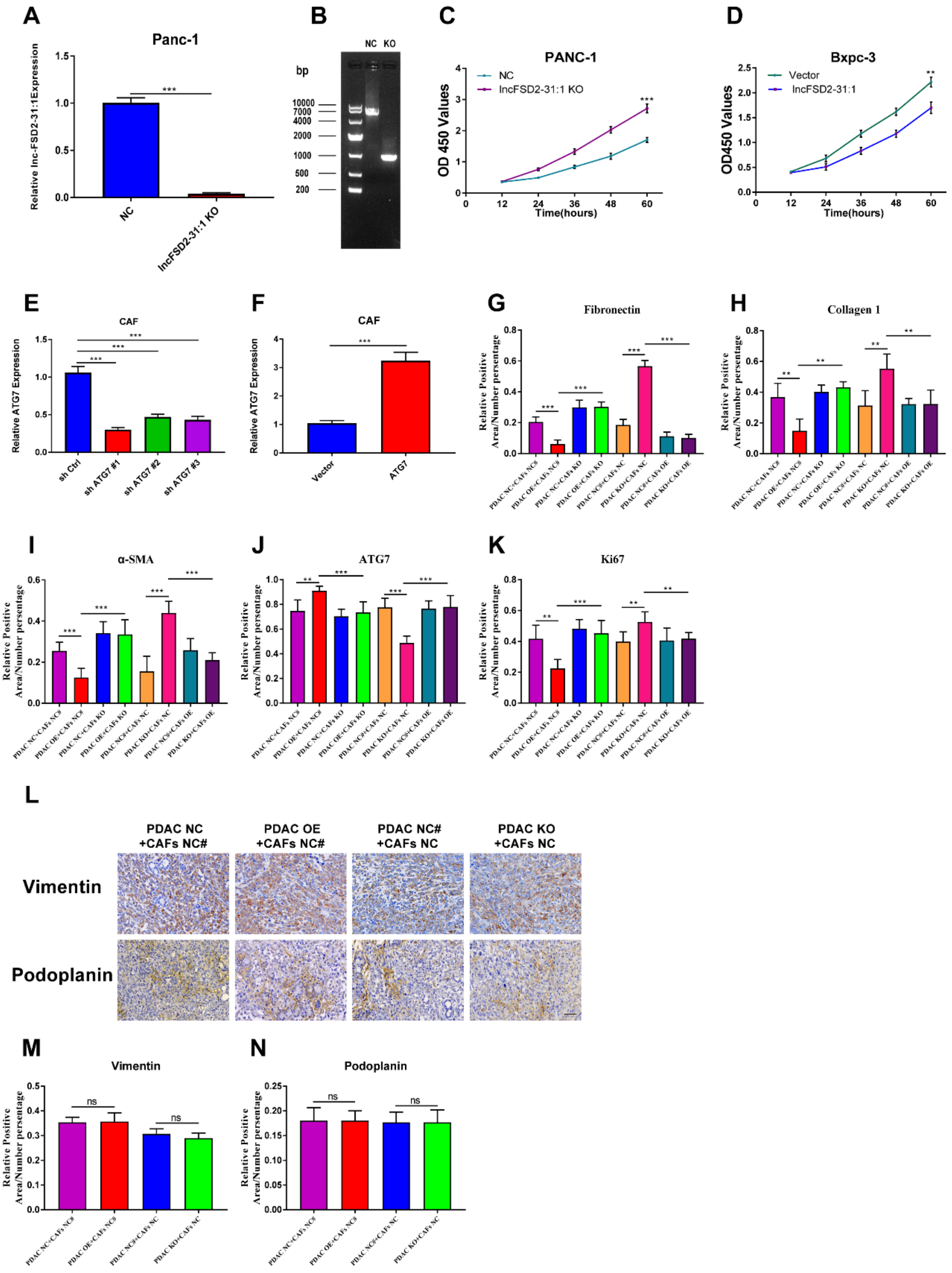

Figure 5. Lnc-FSD2-31:1 inhibits PDAC growth in vitro and *in vivo*. A-B) qRT–PCR

assays shows that lnc-FSD2-31:1 is knocked out by CRISP-Cas9 technology. C-D) The proliferative viability is determined using CCK-8 assays in PANC-1 and BxPC-3 cells with different lnc-FSD2-31:1 expression levels. E-F) ATG7 is effectively silenced/overexpressed by lentiviruses scramble shRNA against ATG7 (LV- sh ATG7) and lentiviral vector encoding ATG7 (LV- ATG7) in CACs through lentivirus transfection as well as their controls. G-K) The expression of Fibronectin, Collagen 1, α-SMA, ATG7, and Ki67 was detected by IHC in orthotopic tumor specimens (original magnification, 20×, scale bar, up 50 μm). The images are scored, and the percentage of positive area/cells is estimated using ImageJ software. L-N) The expression of vimentin and podoplanin is detected using immunohistochemistry in orthotopic tumor specimens (Lnc-FSD2-31:1 NC *vs* OE/KO PANC-1 cells with CAFs) (original magnification, 20×, scale bar, up 50 μm). The images are scored, and the percentage of positive area/cells is estimated using ImageJ software. Statistical significance is assessed by Student's *t*-test. Data are shown as the mean ± SD of three replicates; $**P < 0.01$; $***P < 0.001$.

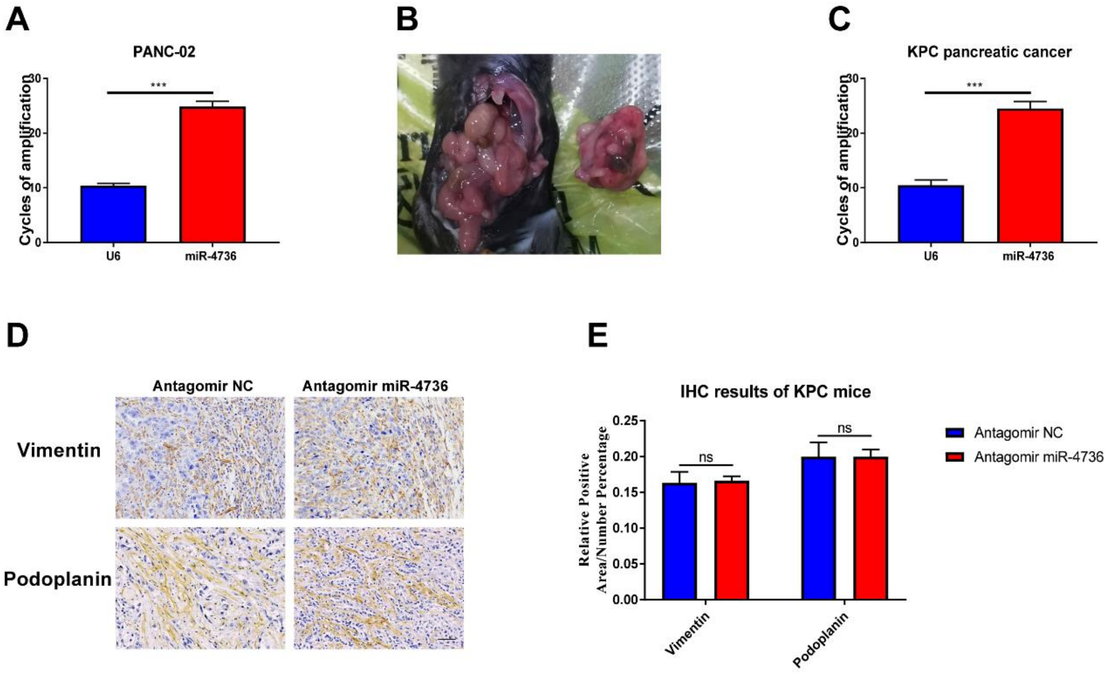

Figure S6. MiR-4736 might be a potential therapeutic and predictive target of PDAC. A-C) qRT–PCR shows the expression of miR-4736 in the mouse pancreatic cancer cell lines PANC-02 and KPC transgenic mouse pancreatic cancer. D-E) The

expression of vimentin and podoplanin is detected by immunohistochemistry in KPC mice (original magnification, 20×, scale bar, up 50 μm). The images are scored and estimated as the percentage of positive area/cells using ImageJ software. Statistical significance is assessed by Student's *t*-test. Data are shown as the mean ± SD of three replicates. ***$P < 0.001$.

Sequence of lnc-FSD2-31:1

GAAGAAACAGCAAAAGGGGAAGTGGGAGAAACAGTAAGGAAGCGTTTTT
AATTAAAAATATGTATACCTAAGAGGAGACTTTATTTTCTATTTCTGGTGGTG
GTGATCTATGTTCTGGAATCCATTTACCAGAAGTTTGTGGATCTTTTGTTTG
AAGTGGAATTGTTCAGTGCCCTACTCTCTTTGACGGTGCAGAGCCTTGCGG
CAAAGTGTTCTCCTGGGCCCTAGAGACCCCCAGGGCACCTGGCAGGAGCC
CGTAAACCACGGCAGAGTCCCAAGTGGTGCATGTTTTGGCGAGTGATGGG
CAGGACTGATGATTCACAGCTGTGAGGCTGTGCTATTGTTTTTACCCTCTTG
TACTGCTTTTAATAGGAAGTCCCGACTTTACGTCAGTTGTGAGAGCAGCGT
CTTATGATGTGACAGGCAAGAAGACATTCCTTGCATCAAATATAAGAGTTTA
ATGAGAAGAGTGGAGTGGATAAGTGAACTCTCCCAAGAGTACTGTTTTTTG
TTTCCCTTTTTAAATGCTTTCTTGGTACATAAATAATATAATACTTTTTTTAAA
GAAATTCCAGATGAGTGGGAAAAAAACCAATCACCTGTTATTACACTATCT
GATTATAACCACTGTTAACATTTCAGTTTTTATCTTTTCAGACTTTTTTCTATG
CATCTGTGTGTGTTTTATTACTTTTTCCATGAAATATAATCATATTATACATAC
TGTTTTATAACTTTTATCCCTTTTAATGCTATATTGTGAATAGATTTCTATATCA
GTAAATATGTAATTACATTATTTTTAACCTCTATGTAGGTTGTACCTTGGATAC
TTAGGTTGTTTCCAATTTTTTTACTGTTGTAGTCAAGGACATAATGAAGTTC
TTCTAACATCCTTGCGAACTTTTCAGAATATTGACTTAGGGTAATTTTCTGA
AGTTATCACTGGCTCAAAAGTACGCGCCTCCCTATCTGCGGCTCCTTTCCTT
CTGAGGCATACAGGACTTCACCCTCCATCCCCCTTGTACTCATTCAGGGTCC
TATGACTAATTCTGGCCAATGAAATGTGAGCACAGGTGACACATGAAGCTC
CAGACAGACACTGAAATGCCCATGAGCAGTTCCGCAGTGTCTGCCTTCCCT
TCCCTCAGCAAATGCTGATGTCCCAGGGACAGAGCCTCTGTCAAGCCTGGA
TCCCTGAGTGACTACGTGGAGCAGCCCCCTTCTCCCTTCATGTTACCCTCAC
CTGCCACCCTGCATGGACGGGTAGCATGAATAAAAAAAAATAAACCTTAGGT
GTTAAACACTGGGGTTTCAGGGTTAATTGGTTAACTGCAGCATAACATATCC
TGTTGCAACAATAAAAAAGAATGCACATTTTTAGGACTTGTAATATGTATTG
CCTCAAATTGCCTTCCAGAAAGACAGACCCAATTATACTCCCACCCACAGT
GTGTGAGTGTGTACTGCTTCCTCTTTAACACTGGAGTTACCCTGGGGACAG
AGAGCTAAAAAAAATATTTTTTTGGTTAAAATGTAACCCTTTAAGGTGTTCA
GCAGTATCATTGATTTCTAAGAGGTTTATTTCATAAACATTGTAAAACAAAA
TTTTAAGTTGCACGTATGGCTTTTTAAAAACGTTTAGACATTTAAAAAAACA
CCTTTTAGTCAAGTTTATCTGTTACTTTTTTTTTTTTTTTTAATGGTTGCTACC
TATGCCGTCATGCTTATGCAGGTCTTTCCAATTCTTTTTTTTTTTTTTTTTTT

TTTTTTTTTTGAGACGGGGTCCACCCTGGCCCCCAGGCTGGAGTGCAGTGG
CGTGATCTCAGCTCACTGCAACCTGCGCCTCCCGGGTTCAAGCGATTCTCA
TGTGTCAACCTCCCTCCTGAGTAGCTGGGACTACAAGTGTATGCCACCACG
CCCAGGTAACTTTTTGTTTTTAGTAGAGATGGGTTTCGCCATGTTGCCTAGG
CTGGTCTCGAACTCCTGAACTCAGGCAATCCACACGCCTTGGCCTCCCAAA
GTGCTGGAATTACAGGTGTGAGCCACCAAGCCCGGCGAGGTCTTCCCAATT
CTGATGGCAAGGTTGTATAAATATTCACCACTATCTTATTCTTGTACTTTTATT
ATTTCTTTCTAAACAGTTAAATCTGTGATCTGGCTGGGCGTGTTGGCTCTTG
CCTGTAATCCCAACACTTTGGGTCGCCAAGGTGGAAGGATCACTTGAGTCC
AGGCATTTGAGGTTACAGTGAGCTTTGATGGCACCGGTGCACTCCAGCCTG
GGGAACAGAGCAAGACCCTGTCTCTAAAAAAAATTTTAAAAACCTCTGAT
CTATCTGGAATGTATTTTCTAAATTACCCCAACACTGTTTATTGGGAAGTCTG
TCCTTTATTCTATATGAAATCCCATTTTTGCCATACACCTAGTAGCCCTTCTGT
TGGGTGCCAGGATAAGCACCCTGGCCTTGAGTGTGCTGTGAACTCTTTATTA
TGGGTTGTGATCTTCCTGCACATTCTAGCACTGGTACCACATTGTTTGGATC
GCCATAGCTTTGGACTAAACTTCCCCATTAAACTTTTTCAAAACTTGCCTAG
TATATTGTGCAGGCCGGGCATGGTGGCTCACGCCTGTAATCCCAGCACTTTG
GGAGGCCAAGGTGGATGGATCACCTGAGATCAGGAGTTCGAGACCAGCCT
GGATAACATGGCGAAACCCCATCTCTACTAAAAATACAAAAAAAAAAAAA
AAAAAAAAAAATTAGCTGGCTGTGGTGATAGGAACCTGTAATCTCAGCTAC
TTGGGAGGCTGAGGCAGTGAACCCAGGAGGCAGAGGTTGCAGTGAGCTG
AGATAGCACCAGCCTGGGCAACGGAGCGAGACTCTGTCTCAAAAAAACAA
AAAACAAAACAAAAAAAAAAACCTTCCAAAAGAAGTTTGAGATAATTTTAT
GTTTAGAAAAATTAGAAATTGGATTGAGTAAAATTTACATATTTTACACAAG
AGGACTAACATCTTTATAATATTTAACCTTTCAGTAAATTTATGTTTTATGTTT
CTCAGCCTATCTTTTCTAGTATAGGTGATTTTTTTAGTGTGGTTACTATTGTA
AATGAGATCCTACTACTTTAAAAAATGAAAATTAAATCTATCGCTTTTATGAC
TTTAAAACATTAGACTTATGTAAGTGTAAACATTATTTAAAAACATAATGTAG
AAAATTAAAGTCCCCCATAATTAAAATCCCTTTCCCCCAGAAAAAGTCAAC
AGCATATTCATCAGTTTTTTCCTAAAGTATGTTTTAGCACTTTGACCATATGA
GACAACCCTTCTGCTGTTAGCATTTTTCTCTTTGCAATAGATCTTCCTATGTC
AGTACACATCAATCTACCTCATTCTTACCTCTGCATAAGAGAGTAGTGTGTA
GTTGTATAATAGTTTAACTTGGCGATTGGTTATTGATTTTGTAACTAGCCAGT
TACTGAATTATTCTAAAGGTTTTGCTGTTGTTAATTTCTTTCGCCTTTTTCAG
TTGGGTAATAGTTCTGGGTAGGTTGATAATTTTATTCCCATATTTGTAATCTTA
TTTCACTTTCTCAACTCATCACATTGACTGGTACTTTTAGAACAATGTTAAA
AAACATTTACATAATTGTGCTGATATTGTACATTATTTGATATATATATATCAA
ATTTTTTTTACTTAGAAGAGTGTTTCTCCAAAACTATAAATTTGACTCTTAGT
GTAAAATAATCTTAATATTTGAATTATACATTCAGGTTATGCCCTCTATTTCTA
GTTTGATGAAAATATTATTAGTAATGGATGTTGGGGTTTTATTAAATGGCTTT
TTTCCACATCTGTCAAGATGATCAACTGGCTTTTCTCTATTATGACTTTAGAT
GAATCGTTTTAATAGATTTGCTAATGTTAAGACATCTCTGCATTTCTACAAGA
ATCTGATGTAGTCATAGTATACTATTCTTTTAATATACATCTCCTTTCAATGTT
AACAGTGGTTATTTATGATTGGTTGGTCCATAGTAGATTTTTATTTTGTGTTT

CTTATCTGTACTTTCTGATTTTTCTACAAAGATTGTGTATTACTTGTATAATAA
AAATAAGAGCTTTAAATTGT

U6-sgRNA(lnc-FSD2-31:1)*4:
GCTAGCGCTACCGGACTCAGGAGGGCCTATTTCCCATGATTCCTTCATATTT
GCATATACGATACAAGGCTGTTAGAGAGATAATTAGAATTAATTTGACTGTA
AACACAAAGATATTAGTACAAAATACGTGACGTAGAAAGTAATAATTTCTTG
GGTAGTTTGCAGTTTTAAAATTATGTTTTAAAATGGACTATCATATGCTTACC
GTAACTTGAAAGTATTTCGATTTCTTGGCTTTATATATCTTGTGGAAAGGAC
GAAACACCGCGCGCTGGGGACGTTATGGTGTTTTAGAGCTAGAAATAGCAA
GTTAAAATAAGGCTAGTCCGTTATCAACTTGAAAAAGTGGCACCGAGTCGG
TGCTTTTTTACCGGTGAGGGCCTATTTCCCATGATTCCTTCATATTTGCATATA
CGATACAAGGCTGTTAGAGAGATAATTAGAATTAATTTGACTGTAAACACA
AAGATATTAGTACAAAATACGTGACGTAGAAAGTAATAATTTCTTGGGTAGT
TTGCAGTTTTAAAATTATGTTTTAAAATGGACTATCATATGCTTACCGTAACT
TGAAAGTATTTCGATTTCTTGGCTTTATATATCTTGTGGAAAGGACGAAACA
CCGATATTCTTGAGATCGGTACAGTTTTAGAGCTAGAAATAGCAAGTTAAAA
TAAGGCTAGTCCGTTATCAACTTGAAAAAGTGGCACCGAGTCGGTGCTTTT
TTCTCGAGGAGGGCCTATTTCCCATGATTCCTTCATATTTGCATATACGATAC
AAGGCTGTTAGAGAGATAATTGGAATTAATTTGACTGTAAACACAAAGATAT
TAGTACAAAATACGTGACGTAGAAAGTAATAATTTCTTGGGTAGTTTGCAGT
TTTAAAATTATGTTTTAAAATGGACTATCATATGCTTACCGTAACTTGAAAGT
ATTTCGATTTCTTGGCTTTATATATCTTGTGGAAAGGACGAAACACCGAAGC
AGAATACTACAACTCCGTTTTAGAGCTAGAAATAGCAAGTTAAAATAAGGC
TAGTCCGTTATCAACTTGAAAAAGTGGCACCGAGTCGGTGCTTTTTTGGAT
CCGAGGGCCTATTTCCCATGATTCCTTCATATTTGCATATACGATACAAGGCT
GTTAGAGAGATAATTGGAATTAATTTGACTGTAAACACAAAGATATTAGTAC
AAAATACGTGACGTAGAAAGTAATAATTTCTTGGGTAGTTTGCAGTTTTAAA
ATTATGTTTTAAAATGGACTATCATATGCTTACCGTAACTTGAAAGTATTTCG
ATTTCTTGGCTTTATATATCTTGTGGAAAGGACGAAACACCGTAAAAAGGC
ACTACTAGATTGTTTTAGAGCTAGAAATAGCAAGTTAAAATAAGGCTAGTCC
GTTATCAACTTGAAAAAGTGGCACCGAGTCGGTGCTTTTTTCTGCAGTCGA
CGGTACCGCGG

qRT-PCR of lnc-FSD2-31:1:
Forward: 5’- ATGAGCACAGCCTTGATGGTG-3’,
Reverse: 5’- ATGGCATGTAACTTAAGAAGGTGG-3’;

Sequence of plasmid using in Dual Luciferase reporter array
ATG7-WT:
GATGGCCCCGCTGTGGGGCTGACTTCTCCCCGGCCGCCTGCTGAGGAGCTC
TCCATCGCCAGAGCAGGACTGCTGACCCCAGGCCTGGTGATTCTGGGCCCC
TCCTCCATACCCCGAGGTCTGGGATTCCCCCCTCTGCTGCCCAGGAGTGGC
CAGTGTTCGGCGTTGCTCGGGATTCAAGATACCACCAGTTCAGAGCTAAAT

AATAACCTTGGCCTTGGCCTTGCTATTGACCTGGGACTTGGTCCTCCATGCA
GTTTTTATTTCTTGTCACAGTGACTGATAGCCATCCCCCAGGATCCTTTCCC
CTTGGCCCTGAGGGGGTGACCCAACACAGACCAAATGGGGAAATGAGCAA
CCAGCTCCTGCCCAGAGCCACTGCGGGAGGTGGCACCCTCATCCCCGGAA
TGTGCTGCCCACCGCACCGCAGGCTCCTCCTGTGGGGGCCCTGGGCATGG
GTGAGGGTGGGACCCCGTGAGCGCACTGCACCCTGGCCCTGGTGGAGCGG
GAGGAGGAGGAGAGCCGAGCTGGGTACGAGACTAAAGGGCCCACATGAC
CCAGTGACGCCAGATTTCCACCAAGGACTGAGTGAGCTGCTCAGACATGG
CTTTCTGCCTCCCAGCCTGTCCTCCACTGTGGGCATAGCATCTGTGCCTGCC
TGCCTGCTTGAGGGAGAGGAGTTTCTGCTGCTGCCTTGAGCTGGGGGGAA
GAGCCCAGGGGCAGATCCTGGCAGCTGCCTGGATGGGGCTCCTCCCTGCC
CTTATGAGCAGGCCAGGCCCAGAAAGGCCGAGCCTGGGCTGCCTTCCTGC
CCCAGCCGAGGGAGGGGTCAGACGGCTCTACCATGGGTAACTCAGGCAAG
AGCTGGTTTTCCTCTTTATTCTGGGTGTGTGCAGCTGTGAGGCCCCAACCC
AGGAGAGGCCATGGCCTAGGTACCTGTGACCACCCTGCCCCCGTGTAGAG
GGCATCGTCTTTCCTGCTATTTTATTCTTTCAGCTTTTGTCTTAGGCCCAGAA
TCAAAGTGAAAATTGAGTCGAGCTGACCCTTACAACAGTAGGATTTAGTAG
GGTAGATTTCAAATGAGGCTTCGCTTCTCCCAAAGTAGCCAGTCCAAGTTC
CAGTGGCTGTCGTTCAGCTCATGGGAGCTTCATGGGGACACAGCCGGCAC
AGGTGCAGGGCCCGAGTCCGCCCACCCAGCCTGGCGCTGAAACTGCACAC
GTACACTATGTGGTTTAAGAGCACTTTATTATTGTTCTTAAGGCTACTTTTAA
GTACAAAAAAAGATGGCCTGCCAAACCTTTTTTTTTCTTCTTCCAGGAAAA
ACAGGCCACAGAGAATGGTATATTACAGATTTACACACATGAAGAGAAGGT
CAGAGCGCACTGCAGGCAGCGCGGCTCTGGGAAGAACTTCACGGAGCCC
CTTCTTAGAGCAGGGAGGGGGCTTTCTCAGTGAAATGTTTGGTTTTCTGCT
GCCTCCTCTGCCCCAGGCCCCCCTCCAGGGTACTGCCTATCCCAGATAGGT
CAGTGCACCAGGGACCCGGCCGCCAGCACCGCCGACCCCTCCCAGAGTGA
CGCCCTTGTTCACTGACAAAGAGACCTGTCCCAGGAGTGTCCTCCACCGA
GCCGGTCAGCTGTGGGTGGTTTTCCTGTTACGACGCTCAGTAGCCTGTAGC
AATAACAAACTCGTGGCTATGAATGCAGATGCAGTGTTCTCATAGAATAACT
GTTCCTGCACTTTTACAGACAAATCTACGACAAAAAAAAAGATCAACTTTT
TTTTTCCGAACAACAAAAAAAAATGAATGATTACAATAGGAAAGGGAAAAA
TTAAATAGCTACATATCATTAACAAATTAATGTTCTTCAAAAAATACCTACAA
ATTTCTCTGTACATTCTTTACGCACAGCGTAACGATGGTCTCAAAATCACCC
ATATAGAAAAGTGTTCTCAACGATTTTTCCTACAGAAAATATAGGGGCCTGA
ATGCCAAAGCTTGGAAGCCCAGTACAGTGGGAGTGAAATGTGTGCGGGGC
AAGGAGAAGGGCTTTTCTTTCCTCCACTTTTCAAAGGCCTGCAGCCACTCT
GTGACTACAAGAGCCAGTCCTCCGACCTTTTCACCCAGTGCCAATTTCCAA
AATTCAACAGCTAAAAACTGTAAAACCGGGGGTCATACGGTGTGCAGAGT
CCACAAAGCCTTGCAGGTGAGGTGACCACGCCCACGTCACCTGGTCAGGT
GCCATCGTCGTGAGCCTCTGGTGGGCCAGGTGGGACACAGCACACCCCAG
GGGGAGGGGATAGAAACGCTCATTGACCAAAAAGGAGCAGCTGTGACCTC
CACAGCTGTGTCTGTCATGCTTGCTTCATCTAATTTCTAGTTAGTAGCTATTA
ATATAGCAAATAATAAATGCAGTAATAACAGTATAAAGTCAGAGGAATGTAT

ACTGCCTTGGCCCCAGCGTACGAGGAAGCGTATAAAACACCATATCACAGA
TTGTCTGTCAGTAATCTGCTGTTCAGCCAAGAGAGTTCAAAGGGAGCAGTT
TCTGCATGTAGGGAAGTTGGAAGACACAAACCCCACCTCCCCTGGGAGCT
TGTAACAAAGCAGACAGGGATGCAAAAATAAATGATGTCAGCCTGCAGCC
AAACTCCAGCATCCCACACCGCAGCTGACCCACTGCTCATCGCGAGGGCC
TGCCAGGAGCTGGCCTCCCGCACTACTTGTGAGTAAAGTGAATATCAAATA
CCAATCTTAGAGTACAACTGTACCAGCAGTAAGTATATCTAGGACTGTAACT
GACAAAAATAAACTAATTCTGAAAAGAA

ATG7- Mut1:
GATGGCCCCGCTGTGGGGCTGACTTCTCCCCGGCCGCCTGCTGAGGAGCTC
TCCATCGCCAGAGCAGGACTGCTGACCCCAGGCCTGGTGATTCTGGGCCCC
TCCTCCATACCCCGAGGTCTGGGATTCCCCCCTCTGCTGCCCAGGAGTGGC
CAGTGTTCGGCGTTGCTCGGGATTCAAGATACCACCAGTTCAGAGCTAAAT
AATAACCTTGGCCTTGGCCTTGCTATTGACCTGGGACTTGGTCCTCCATGCA
GTTTTTATTTCTTGTCACAGTGACTGATAGCCATCCCCCAGGATCCTTTCCC
CTTGGCCCTGAGGGGGTGACCCAACACAGACCAAATGGGGAAATGAGCAA
CCAGCTCCTGCCCAGAGCCACTGCGGGAGGTGGCACCCTCATCCCCGGAA
TGTGCTGCCCACCGCACCGCAGGCTCCTCCTGTGGGGGCCCTGGGCATGG
GTGAGGGTGGGACCCCGTGAGCGCACTGCACCCTGGCCCTGGTGGAGCGG
GAGGAGGAGGAGAGCCGAGCTGGGTACGAGACTAAAGGGCCCACATGAC
CCAGTGACGCCAGATTTCCACCAAGGACTGAGTGAGCTGCTCAGACATGG
CTTTCTGCCTCCCAGCCTGTCCTCCACTGTGGGCATAGCATCTGTGCCTGCC
TGCCTGCTTGAGGGAGAGGAGTTTCTGCTGCTGCCTTGAGCTGGGGGGAA
GAGCCCAGGGGCAGATCCTGGCAGCTGCCTGGATGGGGCTCCTCCCTGCC
CTTATGAGCAGGCCAGGCCCAGAAAGGCCGAGCCTGGGCTGCCTTCCTGC
CCCAGCCGAGGGAGGGGTCAGACGGCTCTACCATGGGTAACTCAGGCAAG
AGCTGGTTTTCCTCTTTATTCTGGGTGTGTGCAGCTGTGAGGCCCCAACCC
AGGAGAGGCCATGGCCTAGGTACCTGTGACCACCCTGCCCCCGTGTAGAG
GGCATCGTCTTTCCTGCTATTTTATTCTTTCAGCTTTTGTCTTAGGCCCAGAA
TCAAAGTGAAAATTGAGTCGAGCTGACCCTTACAACAGTAGGATTTAGTAG
GGTAGATTTCAAATGAGGCTTCGCTTCTCCCAAAGTAGCCAGTCCAAGTTC
CAGTGGCTGTCGTTCAGCTCATGGGAGCTTCATGGGGACACAGCCGGCAC
AGGTGCAGGGCCCGAGTCCGCCCACCCAGCCTGGCGCTGAAACTGCACAC
GTACACTATGTGGTTTAAGAGCACTTTATTATTGTTCTTAAGGCTACTTTTAA
GTACAAAAAAAGATGGGGACGGTAACCTTTTTTTTTCTTCTTCCAGGAAAA
ACAGGCCACAGAGAATGGTATATTACAGATTTACACACATGAAGAGAAGGT
CAGAGCGCACTGCAGGCAGCGCGGCTCTGGGAAGAACTTCACGGAGCCC
CTTCTTAGAGCAGGGAGGGGGCTTTCTCAGTGAAATGTTTGGTTTTCTGCT
GCCTCCTCTGCCCCAGGCCCCCCTCCAGGGTACTGCCTATCCCAGATAGGT
CAGTGCACCAGGGACCCGGCCGCCAGCACCGCCGACCCCTCCCAGAGTGA
CGCCCTTGTTCACTGACAAAGAGACCTGTCCCAGGAGTGTCCTCCACCGA
GCCGGTCAGCTGTGGGTGGTTTTCCTGTTACGACGCTCAGTAGCCTGTAGC
AATAACAAACTCGTGGCTATGAATGCAGATGCAGTGTTCTCATAGAATAACT

GTTCCTGCACTTTTACAGACAAATCTACGACAAAAAAAAAGATCAACTTTT
TTTTTCCGAACAACAAAAAAAAATGAATGATTACAATAGGAAAGGGAAAAA
TTAAATAGCTACATATCATTAACAAATTAATGTTCTTCAAAAAATACCTACAA
ATTTCTCTGTACATTCTTTACGCACAGCGTAACGATGGTCTCAAAATCACCC
ATATAGAAAAGTGTTCTCAACGATTTTTCCTACAGAAAATATAGGGGCCTGA
ATGCCAAAGCTTGGAAGCCCAGTACAGTGGGAGTGAAATGTGTGCGGGGC
AAGGAGAAGGGCTTTTCTTTCCTCCACTTTTCAAAGGCCTGCAGCCACTCT
GTGACTACAAGAGCCAGTCCTCCGACCTTTTCACCCAGTGCCAATTTCCAA
AATTCAACAGCTAAAAACTGTAAAACCGGGGGTCATACGGTGTGCAGAGT
CCACAAAGCCTTGCAGGTGAGGTGACCACGCCCACGTCACCTGGTCAGGT
GCCATCGTCGTGAGCCTCTGGTGGGCCAGGTGGGACACAGCACACCCCAG
GGGGAGGGGATAGAAACGCTCATTGACCAAAAAGGAGCAGCTGTGACCTC
CACAGCTGTGTCTGTCATGCTTGCTTCATCTAATTTCTAGTTAGTAGCTATTA
ATATAGCAAATAATAAATGCAGTAATAACAGTATAAAGTCAGAGGAATGTAT
ACTGCCTTGGCCCCAGCGTACGAGGAAGCGTATAAAACACCATATCACAGA
TTGTCTGTCAGTAATCTGCTGTTCAGCCAAGAGAGTTCAAAGGGAGCAGTT
TCTGCATGTAGGGAAGTTGGAAGACACAAACCCCACCTCCCCTGGGAGCT
TGTAACAAAGCAGACAGGGATGCAAAAATAAATGATGTCAGCCTGCAGCC
AAACTCCAGCATCCCACACCGCAGCTGACCCACTGCTCATCGCGAGGGCC
TGCCAGGAGCTGGCCTCCCGCACTACTTGTGAGTAAAGTGAATATCAAATA
CCAATCTTAGAGTACAACTGTACCAGCAGTAAGTATATCTAGGACTGTAACT
GACAAAAATAAACTAATTCTGAAAAGAA

ATG7- Mut2:
GATGGCCCCGCTGTGGGGCTGACTTCTCCCCGGCCGCCTGCTGAGGAGCTC
TCCATCGCCAGAGCAGGACTGCTGACCCCAGGCCTGGTGATTCTGGGCCCC
TCCTCCATACCCCGAGGTCTGGGATTCCCCCCTCTGCTGCCCAGGAGTGGC
CAGTGTTCGGCGTTGCTCGGGATTCAAGATACCACCAGTTCAGAGCTAAAT
AATAACCTTGGCCTTGGCCTTGCTATTGACCTGGGACTTGGTCCTCCATGCA
GTTTTTATTTCTTGTCACAGTGACTGATAGCCATCCCCCAGGATCCTTTCCC
CTTGGCCCTGAGGGGGTGACCCAACACAGACCAAATGGGGAAATGAGCAA
CCAGCTCCTGCCCAGAGCCACTGCGGGAGGTGGCACCCTCATCCCCGGAA
TGTGCTGCCCACCGCACCGCAGGCTCCTCCTGTGGGGGCCCTGGGCATGG
GTGAGGGTGGGACCCCGTGAGCGCACTGCACCCTGGCCCTGGTGGAGCGG
GAGGAGGAGGAGAGCCGAGCTGGGTACGAGACTAAAGGGCCCACATGAC
CCAGTGACGCCAGATTTCCACCAAGGACTGAGTGAGCTGCTCAGACATGG
CTTTCTGCCTCCCAGCCTGTCCTCCACTGTGGGCATAGCATCTGTGCCTGCC
TGCCTGCTTGAGGGAGAGGAGTTTCTGCTGCTGCCTTGAGCTGGGGGGAA
GAGCCCAGGGGCAGATCCTGGCAGCTGCCTGGATGGGGCTCCTCCCTGCC
CTTATGAGCAGGCCAGGCCCAGAAAGGCCGAGCCTGGGCTGCCTTCCTGC
CCCAGCCGAGGGAGGGGTCAGACGGCTCTACCATGGGTAACTCAGGCAAG
AGCTGGTTTTCCTCTTTATTCTGGGTGTGTGCAGCTGTGAGGCCCCAACCC
AGGAGAGGCCATGGCCTAGGTACCTGTGACCACCCTGCCCCCGTGTAGAG
GGCATCGTCTTTCCTGCTATTTTATTCTTTCAGCTTTTGTCTTAGGCCCAGAA

TCAAAGTGAAAATTGAGTCGAGCTGACCCTTACAACAGTAGGATTTAGTAG
GGTAGATTTCAAATGAGGCTTCGCTTCTCCCAAAGTAGCCAGTCCAAGTTC
CAGTGGCTGTCGTTCAGCTCATGGGAGCTTCATGGGGACACAGCCGGCAC
AGGTGCAGGGCCCGAGTCCGCCCACCCAGCCTGGCGCTGAAACTGCACAC
GTACACTATGTGGTTTAAGAGCACTTTATTATTGTTCTTAAGGCTACTTTTAA
GTACAAAAAAAGATGGCCTGCCAAACCTTTTTTTTTCTTCTTCCAGGAAAA
ACAGGCCACAGAGAATGGTATATTACAGATTTACACACATGAAGAGAAGGT
CAGAGCGCACTGCAGGCAGCGGCTCTGGGAAGAACTTCACGGAGCCC
CTTCTTAGAGCAGGGAGGGGGCTTTCTCAGTGAAATGTTTGGTTTTCTGCT
GCCTCCTCTGCCCCAGGCCCCCCTCCAGGGTACTGCCTATCCCAGATAGGT
CAGTGCACCAGGGACCCGGCCGCCAGCACCGCCGACCCCTCCCAGAGTGA
CGCCCTTGTTCACTGACAAAGAGACCTGTCCCAGGAGTGTCCTCCACCGA
GCCGGTCAGCTGTGGGTGGTTTTCCTGTTACGACGCTCAGTAGCCTGTAGC
AATAACAAACTCGTGGCTATGAATGCAGATGCAGTGTTCTCATAGAATAACT
GTTCCTGCACTTTTACAGACAAATCTACGACAAAAAAAAAAGATCAACTTTT
TTTTTCCGAACAACAAAAAAAATGAATGATTACAATAGGAAAGGAAAAA
TTAAATAGCTACATATCATTAACAAATTAATGTTCTTCAAAAAATACCTACAA
ATTTCTCTGTACATTCTTTACGCACAGCGTAACGATGGTCTCAAAATCACCC
ATATAGAAAAGTGTTCTCAACGATTTTTCCTACAGAAAATATAGGGGCCTGA
ATGCCAAAGCTTGGAAGCCCAGTACAGTGGGAGTGAAATGTGTGCGGGGC
AAGGAGAAGGGCTTTTCTTTCCTCCACTTTTCAAAGGCCTGCAGCCACTCT
GTGACTACAAGAGCCAGTCCTCCGACCTTTTCACCCAGTGCCAATTTCCAA
AATTCAACAGCTAAAAACTGTAAAACCGGGGGTCATACGGTGTGCAGAGT
CCACAAAGCCTTGCAGGTGAGGTGACCACGCCCACGTCACCTGGTCAGGT
GCCATCGTCGTGAGCCTCTGGTGGGCCAGGTGGGACACAGCACACCCCAG
GGGGAGGGGATAGAAACGCTCATTGACCAAAAAGGAGCAGCTGTGACCTC
CACAGCTGTGTCTGTCATGCTTGCTTCATCTAATTTCTAGTTAGTAGCTATTA
ATATAGCAAATAATAAATGCAGTAATAACAGTATAAAGTCAGAGGAATGTAT
ACTGCCTTGGCCCCAGCGTACGAGGAAGCGTATAAAACACCATATCACAGA
TTGTCTGTCAGTAATCTGCTGTTCAGCCAAGAGAGTTCAAAGGGAGCAGTT
TCTGCATGTAGGGAAGTTGGAAGACACAAACCCCACCTCCCCTGGGAGCT
TGTAACAAAGCAGACAGGGATGCAAAAATAAATGATGTCAGCCTGCAGCC
AAACTCCAGCATCCCACACCGCAGCTGACCCACTGCTCATCGCGAGGGGG
ACGGTGGAGCTGGCCTCCCGCACTACTTGTGAGTAAAGTGAATATCAAATA
CCAATCTTAGAGTACAACTGTACCAGCAGTAAGTATATCTAGGACTGTAACT
GACAAAAATAAACTAATTCTGAAAAGAA

Sequences of shRNA (5’-3’):

sh lnc-FSD2-31:1 #1	CATCTGTCAAGATGATCAA
sh lnc-FSD2-31:1 #2	GGACGGGTAGCATGAATAA
sh lnc-FSD2-31:1 #3	GTTGTACCTTGGATACTTA
si ATG7 #1	GAACGAGTATCGGCTGGAT
si ATG7 #2	GATGTCGTCTTCCTATTGA
si ATG7 #3	ACTCGAGTCTTTCAAGACT

sh ATG7 #1	CCGGGCCTGCTGAGGAGCTC
sh ATG7 #2	TCCATCTCGAGATGGAGA
sh ATG7 #3	GCTCCTCAGCAGGCTTTT

Primers and probes(5'-3'):

	**Forward**	**Reverse**
lnc-BHLHA15-1:1	CCTCACCTTCCTGCCGCCAC	TTGCTGTGAAATCTTTTATTTTTGT
NR_037915	GGCGGTTTTTCGACGCTGG	TCTTCAGGGCTCTGCTTTGAG
lnc-ZKSCAN1-3:2	CACTGGAGCATTCTGGAAGA	GACTGGTTCTGCCTCCTACTT
lnc-PEX11B-2:1	GGATATGTGCCCGTGTGGAT	ATCATACCGCTTTGCCCCAT
NR-109777	TTCTGCCCTTTGTGGGAGTTG	CAGCCTGCAGTTCATTTCCG
lnc-NRG4-1:2	ATGCACGCTGGCTTCTCTAC	CCAGGTTTGCACTGCCATAA
lnc-BCAR1-2:1	CCGTGACCGTTTGGCTCTAA	GCTCCTCATGATCCGACCTG
lnc-FSD2-31:1	CATGAGCAGTTCCGCAGTGT	TTTTTATTCATGCTACCCGTCC
lnc-PCDH8-6:2	TGCAAAGAATCCTTGTGCCTACTTA	GAGACAGAGTCTCACCCTGTTGCCC
NR-004401	GCAATCCTCGAAGGGGAGTC	TTCCCCATGTTCTTCGGCTT
β-Actin	CACCATTGGCAATGAGCGGTTC	AGGTCTTTGCGGATGTCCACGT
U6	CTCGCTTCGGCAGCACA	AACGCTTCACGAATTTGCGT
INS	GCAGCCTTTGTGAACCAACAC	CCCCGCACACTAGGTAGAGA
ATG12	CTGCTGGCGACACCAAGAAA	CGTGTTCGCTCTACTGCCC
ULK3	GAAGGACACTCGTGAAGTGGT	ACAATGTGGGGATGTCGAATG
ATG16L2	TGGACAAGTTCTCAAAGAAGCTG	CCTCAGTGCGACCAGTGAT
ATG7	CGTTGCCCACAGCATCATCTTC	CACTGAGGTTCACCATCCTTGG

Antibodies:

CD63	Proteintech	25682-1-AP	WB
CD81	Proteintech	66866-1-Ig	WB
TSG101	Proteintech	14497-1-AP	WB
Alix	Proteintech	12422-1-AP	WB
Calnexin	Proteintech	10427-2-AP	WB
α-SMA	Abcam	ab7817	IF, IHC, WB, Flow Cyt
Podoplanin	Santa Cruz Biotechnology	sc-59347	IF
Vimentin	Proteintech	10366-1-AP	IF, IHC
Collagen 1	Proteintech	14695-1-AP	WB
Collagen 1	Proteintech	66761-1-Ig	IF
Collagen 1	Cell Signaling Technology	72026	IHC
Fibronectin	Proteintech	15613-1-AP	WB
Fibronectin	Proteintech	66042-1	IF
Fibronectin	Cell Signaling Technology	26836	IHC
ATG7	Cell Signaling Technology	8558	IF, IHC, RIP, WB
LC3	Proteintech	14600-1-AP	WB
p62	Proteintech	18420-1-AP	WB
AGO2	Abcam	ab32381	WB，RIP
pan-cytokeratin	Abcam	ab7753	IF

CD-31	Abcam	ab28364	IF
FAP	Invitrogen	PA5-109215	Flow Cyt
Ki67	Cell Signaling Technology	9449	IHC
β-actin	ZSGB-BIO	TA-09	WB
Dylight 550	BOSTER	BA1135	IF
Dylight 488	BOSTER	BA1126	IF
Dylight 550	BOSTER	BA1133	IF
Dylight 488	BOSTER	BA1127	IF

Drug:

GW4869	MERCK	6823-69-4
Bafilomycin	MedChenExpress	HY-100558
